# Single-cell RNA-seq analyses show that long non-coding RNAs are conspicuously expressed in *Schistosoma mansoni* gamete and tegument progenitor cell populations

**DOI:** 10.3389/fgene.2022.924877

**Published:** 2022-09-20

**Authors:** David A. Morales-Vicente, Lu Zhao, Gilbert O. Silveira, Ana C. Tahira, Murilo S. Amaral, James J. Collins, Sergio Verjovski-Almeida

**Affiliations:** ^1^ Laboratório de Parasitologia, Instituto Butantan, São Paulo, Brazil; ^2^ Instituto de Química, Universidade de São Paulo, São Paulo, Brazil; ^3^ Department of Pharmacology, UT Southwestern Medical Center, Dallas, TX, United States

**Keywords:** parasitology, RNA-seq, RNA sequencing, single-cell sequencing data analysis, Schistosoma mansoni adult worms, long non-coding RNAs, single-cell expression profiles

## Abstract

*Schistosoma mansoni* is a flatworm that causes schistosomiasis, a neglected tropical disease that affects over 200 million people worldwide. New therapeutic targets are needed with only one drug available for treatment and no vaccine. Long non-coding RNAs (lncRNAs) are transcripts longer than 200 nucleotides with low or no protein-coding potential. In other organisms, they have been shown as involved with reproduction, stem cell maintenance and drug resistance, and they tend to exhibit tissue-specific expression patterns. *S. mansoni* expresses thousands of lncRNA genes; however, the cell type expression patterns of lncRNAs in the parasite remain uncharacterized. Here, we have re-analyzed publicly available single-cell RNA-sequencing (scRNA-seq) data obtained from adult *S. mansoni* to identify the lncRNAs signature of adult schistosome cell types. A total of 8023 lncRNAs (79% of all lncRNAs) were detected. Analyses of the lncRNAs expression profiles in the cells using statistically stringent criteria were performed to identify 74 lncRNA gene markers of cell clusters. Male gamete and tegument progenitor lineages clusters contained most of the cluster-specific lncRNA markers. We also identified lncRNA markers of specific neural clusters. Whole-mount *in situ* hybridization (WISH) and double fluorescence *in situ* hybridization were used to validate the cluster-specific expression of 13 out of 16 selected lncRNA genes (81%) in the male and female adult parasite tissues; for one of these 16 gene loci, probes for two different lncRNA isoforms were used, which showed differential isoform expression in testis and ovary. An atlas of the expression profiles across the cell clusters of all lncRNAs detected in our analysis is available as a public website resource (http://verjolab.usp.br:8081). The results presented here give strong support to a tissue-specific expression and to a regulated expression program of lncRNAs in *S. mansoni*. This will be the basis for further exploration of lncRNA genes as potential therapeutic targets.

## 1 Introduction

Schistosomiasis is a neglected tropical disease that affects more than 200 million people worldwide (Colley et al., 2014; Mcmanus et al., 2018). Controlling the disease is still a challenge, as no vaccine is currently available (Tebeje et al., 2016; Molehin, 2020). In addition, treatment is restricted to a single drug, praziquantel, which does not act on juvenile worms and against which there are reports of parasite tolerance (Bergquist et al., 2017a; Vale et al., 2017; Kittur et al., 2019). Therefore, the search for new therapeutic targets is needed and understanding the schistosome’s biology on a molecular level could suggest new therapeutic alternatives (Bergquist et al., 2017b).

Single-cell RNA sequencing (scRNA-seq) has been used to advance knowledge of the schistosome’s biology through the identification of specific protein-coding molecular markers of different tissue types in *Schistosoma mansoni* sporocysts (Wang et al., 2018), schistosomula (Diaz Soria et al., 2020) and juvenile/adult worms (Tarashansky et al., 2019; Wendt et al., 2020; Li et al., 2021). Importantly, these works have provided comprehensive protein-coding gene expression cell type atlases at different stages of parasite development. However, the spatial distribution of long non-coding RNAs (lncRNAs) across tissues and cell types has not been assessed yet in *Schistosoma*, even though it is well known that lncRNAs can define cell clusters in other multicellular organisms (Liu et al., 2016; Zhou et al., 2019).

LncRNAs are RNAs longer than 200 nucleotides with low or no protein-coding potential that have been implicated in different biological processes (Ransohoff et al., 2018). They are responsible for a wide range of functions, including regulation of protein-coding gene expression (Jandura and Krause, 2017; Rinn and Chang, 2020) and stem cell maintenance (Chen et al., 2020). Because of their versatile functions and tissue-specific expression, lncRNAs have been proposed as pharmacological targets, especially in human neurodegenerative disorders and cancers (Jiang et al., 2019; Nath et al., 2019).

In *S. mansoni*, we have published a catalogue of thousands of lncRNAs expressed in several stages of the parasite (Maciel et al., 2019), serving as the basis for further studies of these lncRNAs at different conditions. Recently, we have also shown that lncRNAs are potential new therapeutic targets in *S. mansoni* (Silveira et al., 2022). Here, we show for the first time the single-cell landscape of lncRNA distribution across adult *S. mansoni* cell types. We have re-analyzed public scRNA-seq data obtained from *S. mansoni* adult male and immature and mature adult female and identified the lncRNAs signature of schistosome cell types. Analyses of the lncRNAs expression profiles in the cells have identified 74 lncRNA gene markers of cell clusters, many of which were validated with WISH. The results presented here give strong support to a tissue-specific expression and to a regulated expression program of lncRNAs in the parasite, which will be the basis for the exploration of lncRNA genes as potential therapeutic targets in the future.

## 2 Materials and methods

### 2.1 scRNA-seq processing

Single-cell raw fastq files from Wendt et al. (2020) SRA project PRJNA611777 were downloaded via fasterq-dump with the following arguments “-S -e 94 --include-technical”. The integrity of the raw fastq files was checked using vdb-validate, and all files were identified as consistent. To quantify the gene expression of the single-cell data set, we used STARsolo version 2.7.9a (Kaminow et al., 2021) along with a merged gene annotation file containing protein-coding genes, pseudogenes (*Schistosoma mansoni* WormBase gene annotation version 16 (Howe et al., 2017)) and lncRNA genes (Maciel et al., 2019) from a gtf file downloaded from http://verjolab.usp.br/public/schMan/schMan3/macielEtAl2019/files/, along with the genome assembly Smansoni_v7 from WormBase (Howe et al., 2017) with the following parameters “--soloType CB_UMI_Simple --soloCellFilter EmptyDrops_CR --soloFeatures Gene Velocyto GeneFull --soloMultiMappers EM --soloCBwhitelist barcodes_whitelist”. For all samples except SRX7888067, we used the barcode whitelist from Cell Ranger chemistry V2; for sample SRX7888067, we used the barcode whitelist from chemistry V3. Filtered count matrices for all samples were imported into R (R_Core_Team, 2018) using Seurat v4.0.6.9900 (Hao et al., 2021) and cells were further removed from each matrix when the number of features was less than 500, number of counts less than 1000 and greater than 20,000, and percentage of mitochondrial genes greater than 3%. Matrices from all samples were normalized using the NormalizeData function, and variable features were identified using FindVariableFeatures with the following parameters “selection. Method = “vst”, nfeatures = 2000”. Additionally, we scaled the matrices and found principal components using the functions ScaleData, and RunPCA with the parameters “npcs = 100”. To generate the count matrix of all samples, we used the scRNA-seq integration approach from Seurat (Stuart et al., 2019). For that, we first identified integration features using the function SelectIntegrationFeatures, then the integration anchors were identified using the function FindIntegrationAnchors with the following parameters “k.anchor = 20, dims = 1:78, anchor. features = features, reduction = ‘rpca’” and finally integrated the matrices using IntegrateData function. Then, the integrated matrix was scaled using the function ScaleData, and principal components were identified using the function runPCA with the following parameters “npcs = 100”. A final sparse matrix with 48,094 cells was obtained containing expression data for protein-coding genes, pseudogenes, and lncRNAs; and it was used for the following procedures.

### 2.2 Identification of lncRNA cell markers

To assign cell types to our new scRNA-seq data set, we projected the cell cluster annotation from Wendt et al. (2020) onto our re-analyzed scRNA-seq data set. For that, we retrieved the RDS object from the GEO project GSE146736 and imported it into R as a Seurat object using custom scripts. The Wendt et al. (2020) data set was used as the reference, and our new scRNA-seq data set was used as the query to identify cell anchors between both data sets with the function FindTransferAnchors with the following parameters “dims = 1:80, reference. reduction = ‘pca’”. Then we transferred the cell cluster annotation using the function TransferData with the following parameters “dims = 1:80”. Additionally, we transferred the uniform manifold approximation and projection (UMAP) plot embedding from the reference data set to our scRNA-seq data set. For that, we identified the first two UMAP embedding of the reference scRNA-seq data set with the function RunUMAP with the following parameters “return.model = TRUE, n. neighbors = 36, min. dist = 0.70”, then the embedding were transferred to our scRNA-seq data set using the function MapQuery with the following parameters “refdata = list (celltype = “cell_types”), reference.reduction = “pca”, reduction. model = ‘umap’”.

After the cell annotation was transferred, we performed differential expression analysis among all clusters to identify cell type-specific markers. Normalization of read counts across different cells and different samples is particularly important when single-cell RNA-sequencing data is used for downstream analyses, such as differential expression testing, in which the results are confounded by cellular sequencing depth (Hafemeister and Satija, 2019). Moreover, because lncRNAs are known to be expressed at levels lower than those of protein-coding mRNAs, and because different groups of genes with different levels of expression cannot be normalized by the same constant factor (Hafemeister and Satija, 2019), at this step of the analysis we first performed scaled variance stabilization transformation (Hafemeister and Satija, 2019; Choudhary and Satija, 2022) in our scRNA-seq data set using the function SCTransform with the following parameters “method = ‘glmGamPoi’, vst.flavor = ‘v2’, vars.to.regress = ‘percent.mt’”. Then, we set the transferred cell annotation as the active identity of the cells and ran the function FindAllMarkers with the following parameters “only.pos = TRUE, assay = ‘SCT’, min. pct = 0.25, logfc. threshold = 0.25, densify = TRUE, test.use = ‘bimod’”. To select the lncRNA markers, we considered as differentially expressed those genes with less than 0.05 corrected *p*-value in the Wilcox-test in each cluster, and removed differentially expressed genes with a median cluster expression of less than 1 SCT transformed counts compared to all cells of the data set using custom R scripts; this resulted in a final set of 74 lncRNAs identified as lncRNA markers, which were ranked by expression level within the cluster. The clusters where these 74 lncRNAs were identified as markers are shown with an UpSet intersection plot (Lex et al., 2014).

### 2.3 lncRNA markers selection for validation and primer design

To perform *in situ* hybridization experiments for lncRNA marker validation, sixteen lncRNAs were selected based on the clusters where they were identified as markers, on the existence of only one or a few transcript isoforms per gene in the locus, and on the ability to design a probe that only matched a single locus in the genome. To design primers that amplify sequences unique to each lncRNA, each lncRNA marker sequence was searched against the previously published *S. mansoni* transcriptome (Maciel et al., 2019) and only the lncRNA sequence segment that did not match any other transcript was selected for primer design and sequence amplification and cloning.

Information regarding the Gene_ID, lncRNA Transcript _ID and probe size for the 16 selected lncRNA markers is described at Supplementary Table S1. Notably, two different probes were designed for one lncRNA gene marker (G16045). One of the probes targets SmLINC129748, SmLNCA129749, SmLNCA129752, SmLNCA129753 and SmLNCA129758 transcript isoforms, while the other probe targets transcript isoforms SmLNCA129757 and SmLNCA129759. Pairs of primers to clone all 17 lncRNA marker probes were designed using PrimerQuest Tool provided by IDT Integrated DNA Technologies (https://www.idtdna.com/PrimerQuest/) and are shown in Supplementary Table S2. All cloned lncRNA marker sequences were confirmed with Sanger sequencing.

The sequences of interest were inserted into pJC53.2 (available from Addgene https://www.addgene.org/26536/) that had been previously digested with Eam1105I. The insert orientation was confirmed with Sanger sequencing using T3 or SP6 generical primers, and the *in situ* hybridization probes were synthetized accordingly, using T3 or SP6 RNA polymerase, as previously described (Collins et al., 2016; Wendt et al., 2020).

### 2.4 Whole *in situ* hybridization and imaging

Whole mount colorimetric and fluorescence *in situ* hybridization analyses were performed as previously described (Collins et al., 2016; Wendt et al., 2020). All lncRNA probes were used at 10 ng/ml in hybridization buffer, while probes of tissue/cell specific marker for double fluorescence were used at 50 ng/ml in hybridization buffer. All fluorescently labeled parasites were counterstained with DAPI (1 μg/ml) before being cleared in 80% glycerol, then mounted on slides with Vectashield (Vector Laboratories). Brightfield images were acquired on a Zeiss AxioZoom V16 equipped with a transmitted light base and a Zeiss AxioCam 105 Color camera. Confocal imaging of fluorescently-labeled samples was performed on a Zeiss LSM900 Laser Scanning Confocal Microscope.

## 3 Results

### 3.1 LncRNAs identification in adult worm single-cells

To identify the lncRNAs signature of adult schistosome cell types we re-analyzed the publicly available single-cell RNA-sequencing (scRNA-seq) raw data obtained from adult *S. mansoni* by Wendt et al. (2020), as described in detail in the Methods. Briefly, scRNA-seq reads were mapped to the genome using a complete reference transcriptome, including 10,144 protein-coding (Smp) genes, 10,110 lncRNA genes, and 28 pseudogenes, and the numbers of reads mapped per gene locus (not per gene isoform) were counted. After normalization, a total of 17,429 genes were detected, of which 9388 Smps (92.5% of all Smps), 8023 lncRNAs (79.4% of all lncRNAs) and 18 pseudogenes. Our pipeline recovered 48,094 filtered cells, 10.2% more filtered cells than the 43,642 filtered cells recovered by Wendt et al. (2020). The mapping statistics including the number of reads mapped per sample and the number of cells recovered per sample are shown in Supplementary Table S3.

### 3.2 Transfer of cell cluster ID annotations

**FIGURE 1:**
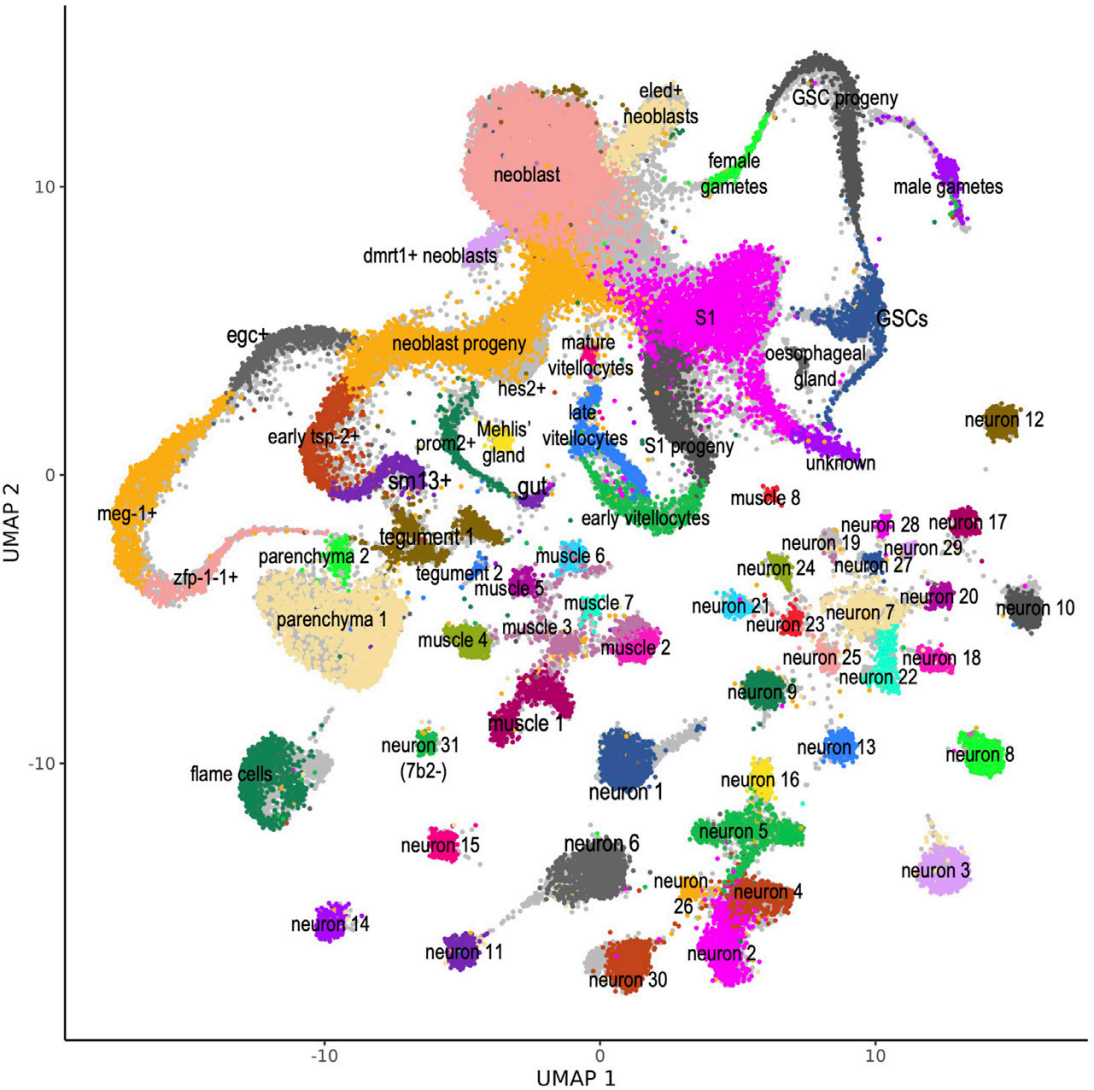
*S. mansoni* atlas of single-cells comprising expression data of protein-coding and lncRNA genes. UMAP plot of the 68 scRNA-seq clusters identified by Wendt et al. (2020) and projected onto our re-analyzed scRNA-seq data set. For each of the 48,094 cells recovered in our re-analysis, expression data for protein-coding and lncRNA genes was used, and all cells were assigned to one of the 68 clusters (see Methods). Cells are colored according to the cluster where they were mapped. Original cell cluster mapping data from Wendt et al. (2020) is shown in the background, colored in light grey. This atlas is available as a public website resource http://verjolab.usp.br:8081.

In the work of Wendt et al. (2020) the single-cells were grouped according to the expression profile of protein-coding genes into 68 different cell clusters, whose identities have been established by determining gene markers specifically expressed in each cluster. In addition, a thorough validation of the specific expression of a given marker at a given adult worm tissue had been obtained with whole mount *in situ* hybridization (WISH) and double fluorescence *in situ* hybridization (dFISH) (Wendt et al., 2020). In order to transfer the cluster annotations to the re-analyzed single-cell set, we used the approach of Stuart et al. (2019) and queried the reference set of cells previously clustered by Wendt et al. (2020) with the newly obtained single-cell expression profile which included lncRNAs in addition to protein-coding genes. With this approach, correspondences between cells in the query and reference datasets can be identified, “anchors” can be used to harmonize datasets into a single reference, and reference labels and data can be projected onto the query dataset (Stuart et al., 2019). To give a visual sense of the cell cluster remapping efficiency we transferred the UMAP embedding from the reference data set to our reanalyzed scRNA-seq data set; Figure 1 shows the cells colored according to the clusters where they were remapped to, and the original cell cluster annotations are shown in light grey in the background. On a few clusters such as flame cells (at the bottom left), which gained approximately 7.5% more cells, or neuron 1, 6 and 30 (at the bottom center) which gained 10–12% more cells, the remapped cells (colored) have clustered more densely than in the original reference data set, leaving some light grey areas visible.

**FIGURE 2:**
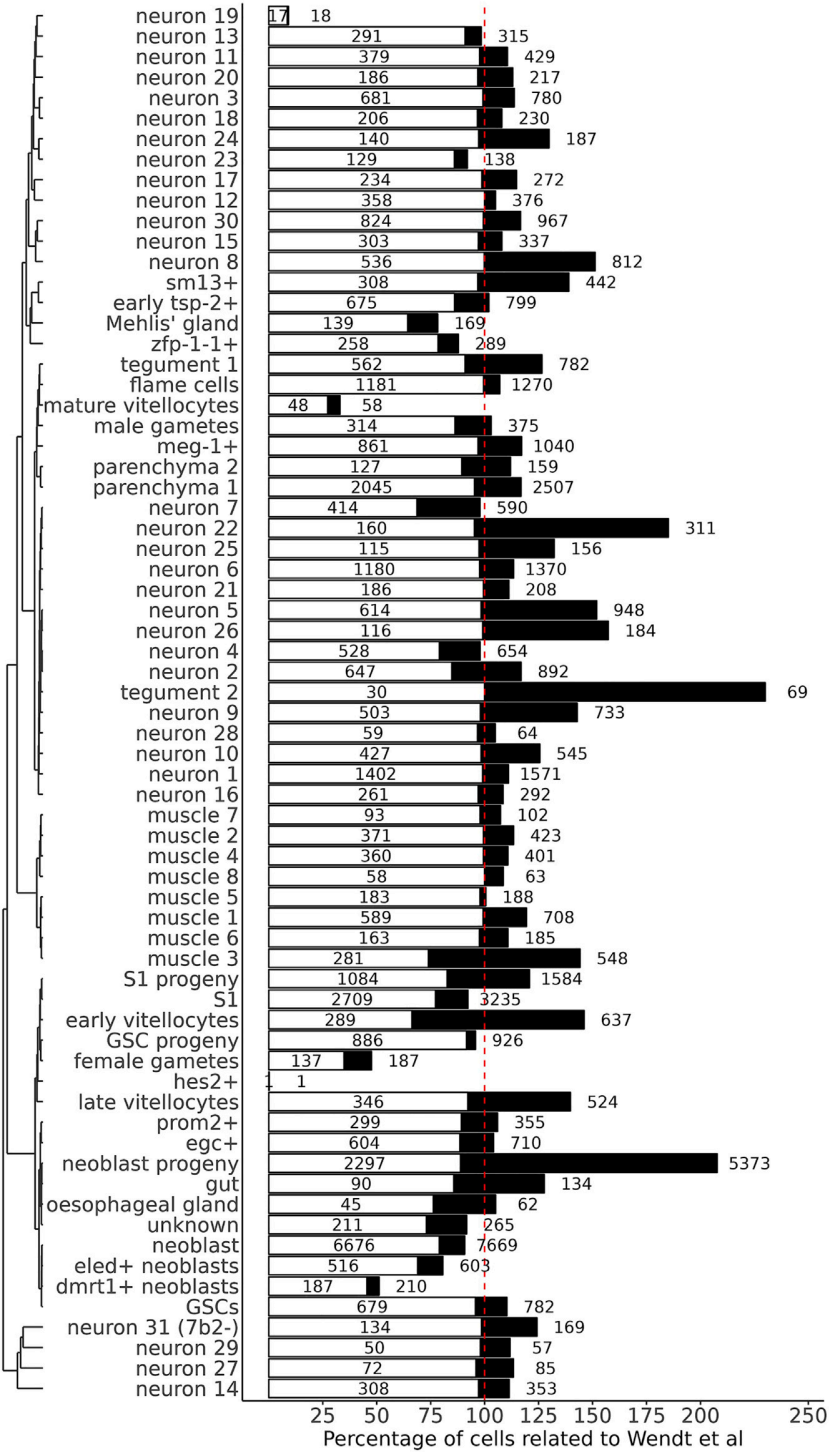
Percentage of cells mapped to each cluster in comparison to the number of cells in the original cluster annotation. The 68 clusters (indicated at left) are grouped according to the similarity of their gene expression patterns in the new, re-analyzed expression data set. The white bars indicate the percentage of cells that remained in the same cluster in the re-mapped data set, relative to the number of cells in the original cluster annotation. The black bars indicate the final percentage of cells in the cluster in the re-mapped data set, relative to the number of cells in the original cluster annotation. The numbers inside the white bars are the absolute numbers of cells that remained in the clusters after re-mapping, and on the right of the black bars are the absolute numbers of total cells in the clusters in the new, re-mapped data set. The vertical dotted red line goes through the 100% value in the *x*-axis.

The percentage of cells mapped to each cluster in comparison to the number of cells in the original cluster annotation of Wendt et al. (2020) is shown in Figure 2. For most of the previously annotated clusters (62 out of 68, i.e. 91%) between 66 and 100% of the cells in the cluster were re-mapped to the same original clusters (Figure 2, see Supplementary Table S4). Note that 44 out of the 62 clusters (i.e. 71%) have between 90 and 100% of the cells coincidentally mapped to the same original clusters (Figure 2, see Supplementary Table S4). Only 6 clusters had less than 66% of the cells coincidentally mapped to the same original clusters; the cluster in which most of the cells were transferred to other clusters was the hes2^+^, where 531 out of its 561 cells (94.7%) were transferred to the neoblast progeny cluster and 13 cells (2.3%) to neoblast cluster (Supplementary Table S4), followed by the neuron 19 cluster, where 177 out the 198 cells (89.4%) were transferred to the neuron 8 cluster. The other four clusters which lost a considerable fraction of the original cells were dmrt1+ neoblasts, where 189 out of 409 cells (46.2%) were transferred to the neoblast cluster; female gametes, where 155 out of 388 cells (39.9%) were transferred to neoblast; mature vitellocytes, where 59 out of 154 cells (38.3%) were transferred to neoblast progeny; and Mehlis’ gland, where 64 out of 214 cells (29.9%) were transferred to neoblast progeny and neoblast clusters (Supplementary Table S4). The median gene expression in the same set of cells, grouped by the original cell cluster annotation, were highly similar (Pearson correlation = 0.97–1.00) between the Wendt et al. (2020) reference matrix, which had only protein-coding genes, and the new query matrix, which includes lncRNAs (Supplementary Figure S1A), thus ruling out the possibility of transfer mislabel due to difference in the gene count strategies. A possible explanation for the loss of cells from one cluster to another is the overall similarity between many of the Wendt et al. (2020) clusters, as documented by the correlation coefficient between the median expression of clusters in the reference matrix (Supplementary Figure S1B). One good example of this is the hes2^+^, a subcluster of neoblast progeny, that lost most of its cells to the neoblast progeny cluster; both clusters are highly similar as determined by the Pearson correlation (0.973) (Supplementary Figure S1B).

Our filtering and mapping strategy (see Methods) was able to recover 5039 new, previously non-identified cells, of which 884 (17.5%) were mapped to the neoblast progeny cluster, followed by 394 cells (7.8%) mapped to the parenchyma 1 cluster, and 262 cells (5.2%) mapped to the neuron 5 cluster (Supplementary Table S4, orange). The remaining previously non-identified cells were mapped at different smaller extents (4.4–0.02%) to 64 other clusters (Supplementary Table S4).

### 3.3 Identification of lncRNA markers of cell clusters

**FIGURE 3:**
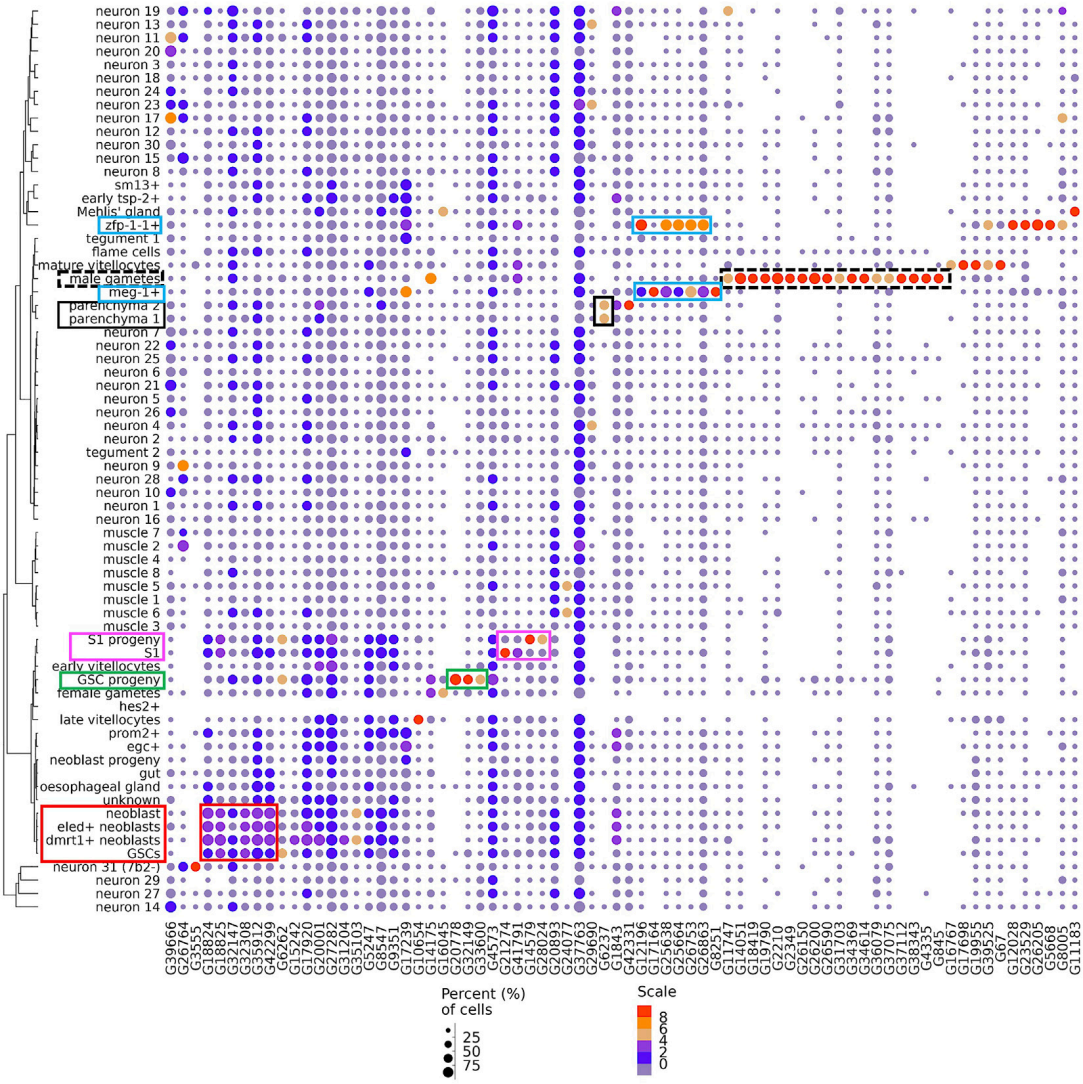
Dot-plot of 74 lncRNA genes identified as markers of single-cell clusters. A dot-plot summarizing the cluster-specific expression of each of the 74 lncRNA genes identified as markers of cell clusters in adult *S. mansoni*. Cluster IDs are on the vertical axis and gene IDs are on the horizontal axis. Expression levels are colored by gene expression (blue = low, red = high). Percentage of cells in the cluster expressing the gene is indicated by the size of the circle (small = few, large = many). The colored boxes highlight the lncRNAs cited in the Results.

To find lncRNA markers of cell clusters we looked for lncRNAs which were significantly more highly expressed in one cluster compared with all other clusters. For this, we applied a variance stabilization transformation of the data (see Methods) using the “regularized negative binomial regression” statistical approach (Hafemeister and Satija, 2019) to remove from the downstream analyses the influence of scRNA-seq technical characteristics such as sequencing depth, while preserving biological heterogeneity. Subsequently, a differential expression analysis among all clusters identified 74 lncRNA genes as markers of cell clusters (Figure 3).

A set of 18 lncRNAs were conspicuously expressed in male gametes (Figure 3, dotted black box). A total of 7 lncRNAs were expressed in the meg-1^+^ cluster (Figure 3, blue box), and 5 of them were also expressed in the zfp-1-1^+^ cluster, two clusters that belong to the tegument lineage.

The 3 lncRNAs at the left-most end of the image (Figure 3) were expressed in a number of different neuron clusters, and the next 6 lncRNAs to the right of those were more highly expressed in germline stem cells (GSCs) and neoblasts (Figure 3, red box), clusters of progenitor cells for gametes and somatic tissues, respectively. Three lncRNAs (G20778, G32149 and G33600) were highly expressed only in the GSC progeny cluster (Figure 3, green box), while four lncRNAs were markers of S1 progeny and S1 (Figure 3, magenta box), two of them (lncRNAs G14579 and G28024) in the S1 progeny cluster and two (G21274 and G41791) in the S1 cluster. Interestingly, lncRNA G6237 was detected as expressed only in the parenchyma 1 and parenchyma 2 clusters (Figure 3, black box).

**FIGURE 4:**
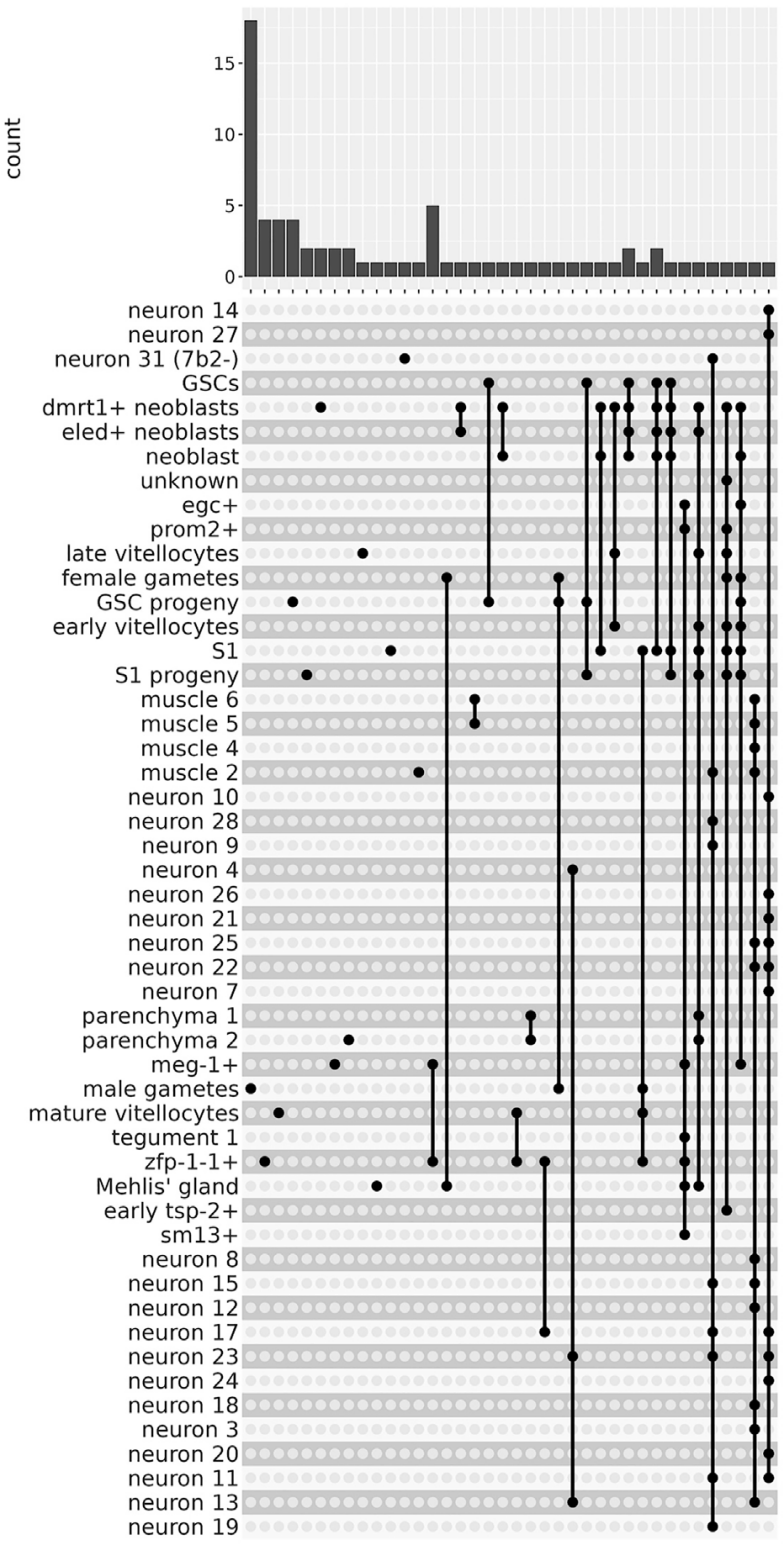
The lncRNAs are markers of 51 different single-cell clusters. The UpSet intersection diagram shows on the upper panel the number of *S. mansoni* lncRNAs (*y*-axis) that have been detected in each of the intersection sets, indicated by the connected points in the lower part of the plot, as being markers of the indicated single-cell clusters. On the left-most part of the plot are the lncRNAs that are markers of a unique single-cell cluster. On the right-most part are the lncRNAs that are markers of a group of single-cell clusters, joined with the connected dots.

There were 13 clusters in which we were able to identify sets of lncRNAs that were significantly more highly expressed exclusively in a single cluster (Figure 4, left-most side). For example, male gametes cluster had 18 exclusive lncRNA markers (Figure 4 top panel, see Supplementary Table S5 for lncRNA gene names); zfp-1-1^+^, mature vitellocytes and GSC progeny clusters had 4 exclusive lncRNA markers each, and another 9 clusters had one or two exclusive lncRNA markers each (Figure 4 top panel; see Supplementary Table S5). Besides those lncRNAs exclusively more expressed in a single cluster, we identified lncRNA markers that were shared by two or more cluster groups (Figure 4); one such interesting example is lncRNA G39666 that was a marker of 13 different neuron clusters (Figure 4, right-most end; see Figure 3, left-most lncRNA). These results are in accordance with the findings in human cell lines (Encode_Project_Consortium et al., 2007), in which the vast majority of intronic and intergenic long non-coding RNA transcripts are expressed only in 1 cell line (out of the 11 cell lines tested), while the majority of protein-coding transcripts are expressed in one up to 7 cell lines (out of the 11) (Encode_Project_Consortium et al., 2007).

### 3.4 Validation of lncRNA neuron markers

We have selected 16 out of the 74 lncRNA gene markers (Supplementary Table S1) to visualize their sites of expression in adult male and female worm tissues with WISH, based on the clusters where they were identified as markers, on the existence of only one or a few transcript isoforms per gene in the locus, and on the ability to design a probe that only matched a single locus in the genome. A total of 13 out of the 16 selected lncRNAs (81%) were validated, as they were found localized in tissues that are consistent with the cell clusters of which they are markers; of these, 9 were also detected at some other tissues. Three, lncRNAs G38343, G20001, and G17920 were detected in tissues not consistent with the scRNA-seq analysis (see Supplementary Table S1). All of them are described below.

**FIGURE 5:**
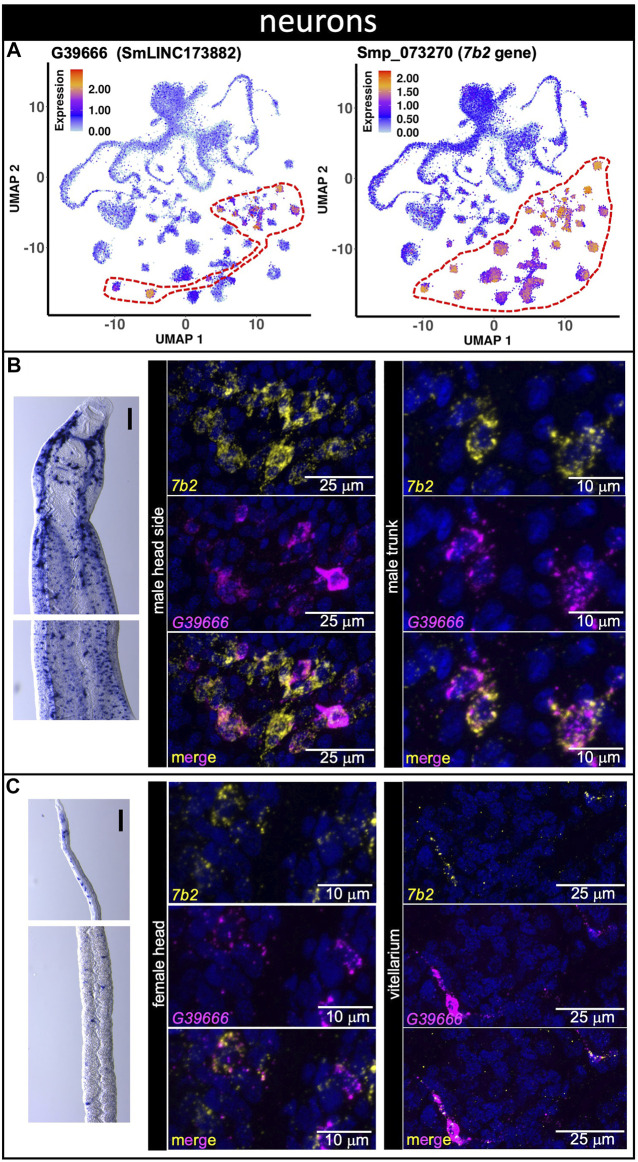
A lncRNA marker of 13 different neuron clusters co-localizes with neuroendocrine protein *7b2* message. **(A)** UMAP plot of lncRNA neurons cluster marker G39666 (left) and of general neuronal marker *7b2* (right). UMAP plots are colored by gene expression (blue = low, red = high) and the scale represents log10(UMIs+1). The regions enclosed by red dashed lines indicate the location of the relevant neuron clusters on the UMAP plots. **(B,C)** Whole-mount *in situ* hybridization (WISH) of lncRNA gene G39666 in the head (left, top) and body (left, bottom) of a male **(B)** or a mature female **(C)**. Scale bars are 100 µm. Double FISH with G39666 lncRNA and *7b2* of male **(B)** head sides (middle) and trunk (right), and of female **(C)** head (middle) and vitellarium (right). Nuclei: blue.

The lncRNA marker of 13 different neuron clusters, lncRNA G39666 (SmLINC173882) (Figure 5A, see Supplementary Table S5), was detected by WISH in the head sides and body of males (Figure 5B, left) as well as in the head and in proximity to the vitellaria of females (Figure 5C, left) with a pattern that is very similar to that of neuroendocrine protein *7b2* gene (Wendt et al., 2020), a general neuron marker. This result supports our previous study, in which G39666 (SmLINC173882) was present in the turquoise gene co-expression network module involved in generation of neurons, synapse, locomotory behavior and axon guidance (Maciel et al., 2019). In fact, dFISH showed that G39666 and the neuroendocrine protein *7b2* messages co-localized in the head side cells of males and in the trunk (Figure 5B, middle, right), as well as in the head of females and in a few neuron cells near the vitellaria (Figure 5C, middle, right).

**FIGURE 6:**
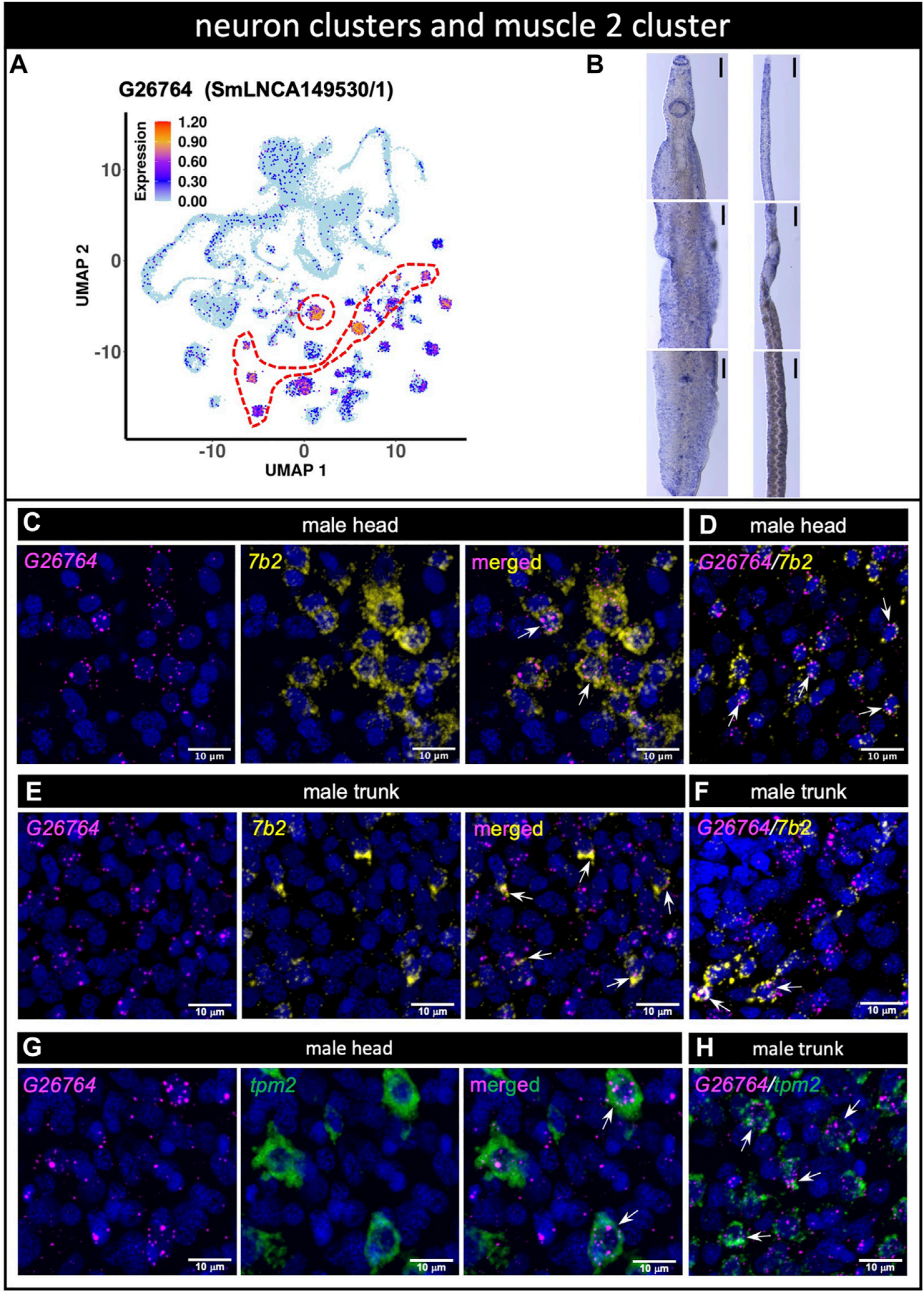
**(A)** lncRNA marker of 8 different neuron clusters and of muscle 2 cluster co-localizes with neuroendocrine protein *7b2* and muscle tropomyosin genes. (A) UMAP plot of lncRNA cluster marker G26764. UMAP plot is colored by gene expression (blue = low, red = high) and the scale represents log10(UMIs+1). The region enclosed by the red dashed line indicates the location of the relevant neuron cluster on the UMAP plot. **(B)** WISH of lncRNA G26764 in the head (top), trunk (middle) and tail (bottom) of a male [left] or a mature female [right]. Scale bars are 100 µm. **(C–F)** Double FISH with lncRNA G26764 and *7b2* gene in male head **(C,D)** and trunk **(E,F)**; panels **D** and **F** show the dFISH images of other worm sections different from **(C)** and **(E) (G,H)** Double FISH with lncRNA G26764 and the general muscle marker gene *tpm2* tropomyosin in the male head **(G)** and trunk **(H)**. Nuclei: blue.

LncRNA G26764 (SmLNCA149530/1) was identified with scRNA-seq as a marker of 8 different neuron clusters and of the muscle 2 cluster (Figure 6A, see Supplementary Table S5). G26764 lncRNA was detected by WISH as dispersed throughout the bodies of males and females (Figure 6B). dFISH showed that the G26764 lncRNA was detected in the head and trunk cells in well-defined spots near the nuclei of cells expressing the neuroprotein *7b2* mRNA (Figures 6C–F). Consistent with the scRNA-seq data, G26764 was detected also in cells expressing the general muscle marker tropomyosin 2 (*tpm2*) (Figures 6G,H).

### 3.5 LncRNAs as markers of reproductive tissues

**FIGURE 7:**
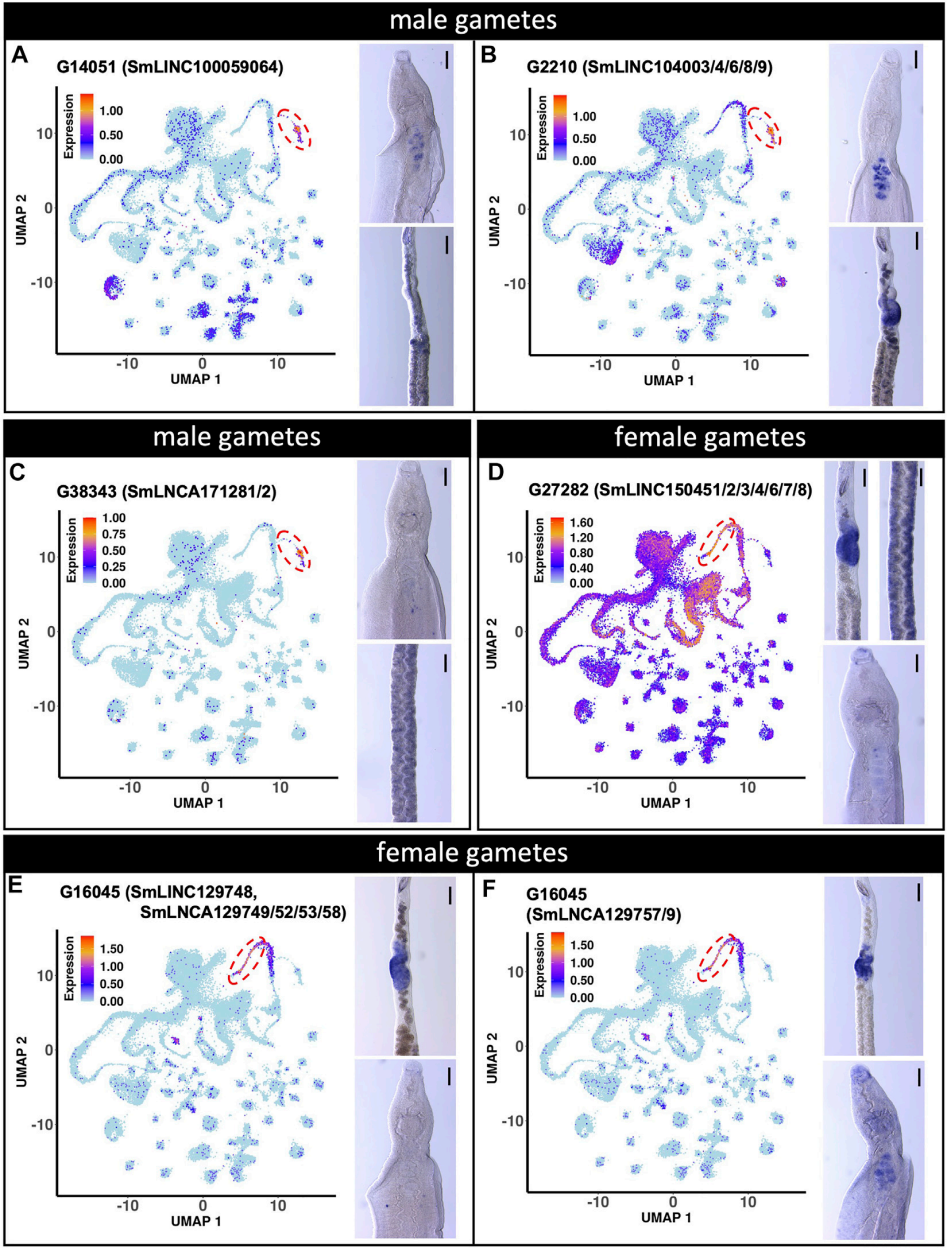
lncRNA markers of male and female gametes single-cell clusters are localized in testis and ovary. **(A** to **C)** UMAP plot (left) of the indicated lncRNA marker of male gametes cluster. WISH with the indicated lncRNA in a male head [right, top] and the ovary region of a female [right, bottom]. **(D** to **F)** UMAP plot (left) of the indicated lncRNA marker of female gametes cluster. WISH with the indicated lncRNA in a female region of the ovary and vitellarium [**D**, right, top] and male head [**D**, right, bottom]. WISH of the ovary region of a female [**E**,**F**, right, top] and male head [**E**,**F**, right, bottom]. UMAP plots are colored by gene expression (blue = low, red = high) and the scale represents log10(UMIs+1). The regions enclosed by the red dashed lines indicate the location of the relevant male or female gametes cluster on the UMAP plots. WISH scale bars are 100 µm.

The expression of three male gamete-enriched lncRNA markers were also evaluated (Figure 7). G14051 (SmLINC100059064) and G2210 (SmLINC104003/4/6/8/9) lncRNAs were confirmed by WISH to be expressed in the male testis (Figures 7A,B). Surprisingly, G14051 expression was also detected by WISH in mature vitellocytes (Figure 7A), despite not being detected in these cells by scRNA-seq. Nevertheless, a re-analysis of publicly-available RNA-seq data ((Silveira et al., 2021), https://verjolab.shinyapps.io/Reference-genes/) showed evidence of G14051 (SmLINC100059064) transcription in female worms, albeit at a level 3 to 6 times lower than in males. Perhaps not surprisingly given its modest level of expression in female germ cells, G2210 was also detected by WISH in the female ovary (Figure 7B). Despite its high-level of expression in male germ cells by scRNA-seq, by WISH G38343 (SmLNCA171281/2) lncRNA showed little expression in male worms but was weakly expressed in cells in or near the vitellaria of female schistosomes (Figure 7C).

We also examined the expression of two female gametes lncRNA markers (Figures 7D–F). G27282 (SmLINC150451/52/53/54/56/57/58) lncRNA expression was confirmed by WISH to be expressed in the ovary (female gametes) (Figure 7D). Interestingly, as suggested by our scRNA-seq analysis, G27282 was also detected by WISH to be expressed in the vitellarium (Figure 7D). The other lncRNA, G16045 had 12 different transcript isoforms detected in the scRNA-seq dataset (http://verjolab.usp.br:8081/cluster_view/G16045). Two different probes were designed for the transcripts in the G16045 gene locus, each representing one of two groups of transcripts. Each group has a common last exon, which is different in the two groups of transcripts in the G16045 gene locus (see this locus in the genome browser, along with mapping of the pairs of primers that were used to generate the two probes). The transcripts in the group SmLINC129748/SmLNCA129749/52/53/58 (Figure 7E) were confirmed by WISH to be expressed in the female ovary and absent from male testis, in accordance with our previously published work, in which SmLINC129748/SmLNCA129752 was expressed in females and almost absent in male worms ((Silveira et al., 2021), https://verjolab.shinyapps.io/Reference-genes/). Interestingly, the other group of transcript isoforms representing SmLNCA129757/9 (Figure 7F) was detected by WISH as expressed in the ovary, and also in male testis, illustrating that a different lncRNA transcript isoform from a single locus can have their expression differentially regulated in different adult worm tissues.

A marker detected with scRNA-seq in the mature vitellocytes cluster, lncRNA G17698 (SmLINC132934, SmLNCA132935/36/37/38/40/41) (Supplementary Figure S2, top) was confirmed by WISH to be well expressed in mature vitellocytes throughout the female bodies (Supplementary Figure S2, bottom). Of note, transcripts in the lncRNA G17698 *locus* were detected as belonging to the female pink gene co-expression module related to endoplasmic reticulum, protein and glycoprotein biosynthetic processes (Maciel et al., 2019), which are functions important for egg production.

### 3.6 Validation of lncRNA tegument markers

**FIGURE 8:**
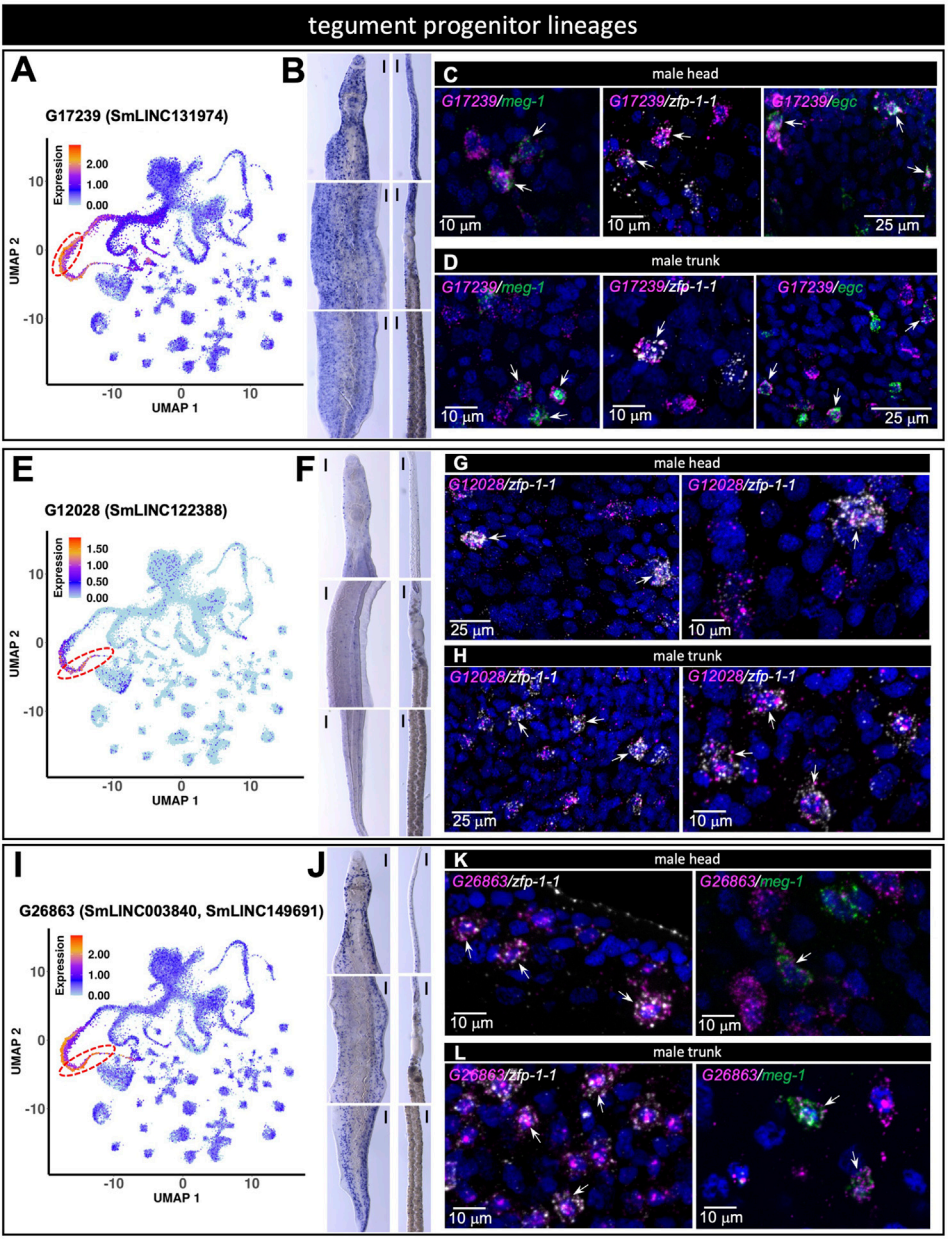
lncRNA markers of tegument progenitor lineages co-localize with protein-coding genes known to mark those tegument progenitors. **(A,E,I)** UMAP plot of the indicated lncRNA marker of tegument progenitor lineages. **(B,F,J)** WISH with the indicated lncRNA in a male [left] and a female [right] head [top], trunk [middle] and tail [bottom]. **(C,G,K)** Double FISH in male head with the indicated lncRNA and the general tegument progenitor marker genes *meg-1*, *zfp-1-1*, *egc* **(C)**, *zfp-1-1* **(G)**, *zfp-1-1*, *meg-1* **(K)**. **(D,H,L)** Double FISH in male trunk with the indicated lncRNA and the general tegument progenitor marker genes *meg-1*, *zfp-1-1*, *egc* **(D)**, *zfp-1-1* **(H)**, *zfp-1-1*, *meg-1* **(L)**. UMAP plots are colored by gene expression (blue = low, red = high) and the scale represents log10(UMIs+1). The regions enclosed by the red dashed lines indicate the location of the relevant meg-1^+^ **(A)** or zfp-1-1^+^ **(E,I)** clusters on the UMAP plots. WISH scale bars are 100 µm.

Tegument progenitor cluster markers were assayed with three probes representing different marker lincRNAs. G17239 (SmLINC131974) was identified with scRNA-seq as a marker of meg-1^+^ cells (Figure 8A) and was detected by WISH throughout the head and body of both male and female worms (Figure 8B). dFISH analysis confirmed the co-localization of G17239 with *meg-1* transcripts in the male head and trunk (Figures 8C,D). We observed similar co-localization patterns of G17239 with *zfp-1-1* and *egc* messages, corroborating the detection with scRNA-seq of G17239 in zfp-1-1^+^ and *egc* ^+^ clusters (Figure 8A). The second tegument progenitor marker, G12028 (SmLINC122388) was identified with scRNA-seq as a marker of zfp-1-1^+^ cluster (Figure 8E), and was detected in small numbers of cells by WISH in the head and body of both male and female worms (Figure 8F); dFISH confirmed that G12028 and *zfp-1-1* messages were co-localized in the head and trunk of males (Figures 8G,H). The third marker, G26863 (SmLINC003840, SmLINC149691) was identified by scRNA-seq as a marker of the zfp-1-1^+^ cluster (Figure 8I) with expression also in the meg-1^+^ cluster. WISH showed expression of G26863 both in the head and trunk of male and female schistosomes (Figure 8J) and dFISH confirmed G26863 co-expression with *zfp-1-1* and with *meg-1* in male head and trunk (Figures 8K, L).

Three additional lncRNA probes were assayed as tegument progenitor zfp-1-1^+^ cluster markers, namely G25638 (SmLINC147486/7/8), G12196 (SmLINC002017/25, SmLINC122706/7) and G26205 (SmLINC148606) (Supplementary Figure S3A–L). The three were similarly detected by WISH throughout the tegumental lineage, as described above, and were confirmed with dFISH to co-localize with *zfp-1-1* message in the male head and trunk, except for G26205, which was detected with dFISH only in the trunk (Supplementary Figure S3A–L). A weighted gene co-expression analysis had previously identified that expression of all these tegument lncRNA markers is correlated with the brown or turquoise co-expression modules involved in focal adhesion, actin cytoskeleton, cell and adherens junctions or contractile fibers (Maciel et al., 2019), which are cellular components consistent with their finding in the tegument.

Finally, Supplementary Figure S4 shows two markers whose localizations were not confirmed. LncRNA G20001 (SmLINC137107), a parenchyma 1 marker identified with scRNA-seq (Supplementary Figure S4A), was detected by WISH in a pattern (Supplementary Figure S4B) that is similar to the pattern of labeling of parenchyma 1 determined by Wendt et al. (2020), however dFISH did not show colocalization with parenchyma marker *tgfbi* (Supplementary Figures S4C-H). LncRNA G17920 (SmLINC133371), was identified by scRNA-seq as a marker of the dmrt^+^ and eled^+^ neoblasts (see Supplementary Table S5). However, we detected no expression consistent with neoblast expression (Supplementary Figure S4H). Instead, by WISH we detected G17920 most highly in the germ cells of male and female worms, which is not unexpected given the detection of G17920 expression in these cell types by scRNA-seq (Figure S4G). The WISH approach depends on the *in situ* accessibility of the probe to the target transcript, which may be more tightly associated with different protein or DNA partners in different tissues, and eventually not available to base-pair with the probe.

### 3.7 lncRNAs detected as expressed exclusively in one cluster

It is known in certain human cell types that the expression of thousands of lncRNAs is more heterogeneous than the expression of mRNAs (Lv et al., 2016; Yunusov et al., 2016), and it has been proposed that averaging transcriptomes over thousands of cells masks the presence of rare cells with high lncRNAs expression (Mercer et al., 2008; Yan et al., 2013). In fact, an analysis of single-cell RNA-seq data from each of five different human cell types (Yunusov et al., 2016) showed that, when comparing on a cell-to-cell basis the lncRNAs to the protein-coding mRNAs that are expressed at similar low levels in a given cell type, there is a statistically significant higher heterogeneity of expression of lncRNAs (Yunusov et al., 2016), possibly reflecting the specific roles played by lncRNAs on different individual cells that are not synchronized among them in a given tissue. Therefore, we postulated that another way of identifying interesting cluster-enriched lncRNAs, possibly important for function in *S. mansoni* adult worms, was to look for lncRNAs that were detected as expressed in only one cluster, and in at least 10% of the cells of that given cluster. This stands as a complementary way to look for cluster-enriched lncRNAs, besides finding lncRNA markers, which are the lncRNAs significantly more highly expressed in a given cluster compared with the median expression in all other clusters.

We found 204 lncRNAs that were detected as expressed in at least 10% of cells exclusively in one cluster among the 68 clusters analyzed in this work, with no other cluster expressing the indicated lncRNA (Supplementary Table S6). Interestingly, male gametes cluster has 55 such exclusive lncRNAs, with G38343 (SmLNCA171281/2), the most frequent one, being expressed in 74% of the male gamete cells. In fact, there are 10 lncRNAs expressed in more than 50% of male gamete cells (Supplementary Table S6), with the remaining 45 lncRNAs being expressed in the range of 45 to 10% of the cells. Female gametes cluster has 10 lncRNAs exclusively expressed in more than 10% of cells, the most frequent one, G29240 (SmLINC154048) being expressed in 32% of the female gamete cells (Supplementary Table S6). Late vitellocytes has 16, and mature vitellocytes has 9 lncRNAs exclusively expressed in more than 10% of cells; the most frequent in late vitellocytes is G25294 (SmLNCA146832), expressed in 42% of the cells, and in mature vitellocytes the most frequent is G17698 (SmLINC132934 to SmLINC132941), expressed in 57% of the mature vitellocyte cells (Supplementary Table S6).

Noteworthy, the tegument progenitor zfp-1-1^+^ cluster has 14 lncRNAs, and the tegument 1 cluster has only one lncRNA, G29145 (SmLINC153880/1/3) exclusively expressed in more than 10% of cells (Supplementary Table S6). Interestingly, G29145 was found to be expressed in 25% of cells in the tegument 1 cluster, and in no other cluster it was expressed in at least 10% of cells. When observed in the sub-sets of scRNA-seq data (http://verjolab.usp.br:8081/cluster_view/G29145) G29145 showed a sex-specific expression, being detected only in the tegument 1 cluster of immature and mature females, with no expression detected in males.

We observed that for each cluster of cells analyzed in this work, when looking at all expressed genes, not necessarily exclusively detected in any cluster, there was a correlation between the level of expression of the genes and the fraction of cells from the cluster in which the genes were detected, both for lncRNAs and mRNAs (Supplementary Figure S5A, B), which indicates that the depth of RNA-sequencing might impact the frequency of detection of lowly expressed genes. Nevertheless, we observed many conspicuous outliers that were expressed at high levels and yet were detected in only 10–30% of the cells (Supplementary Figure S5), suggesting that they were genes that could play a specific role in a fraction of cells in that cluster.

**FIGURE 9:**
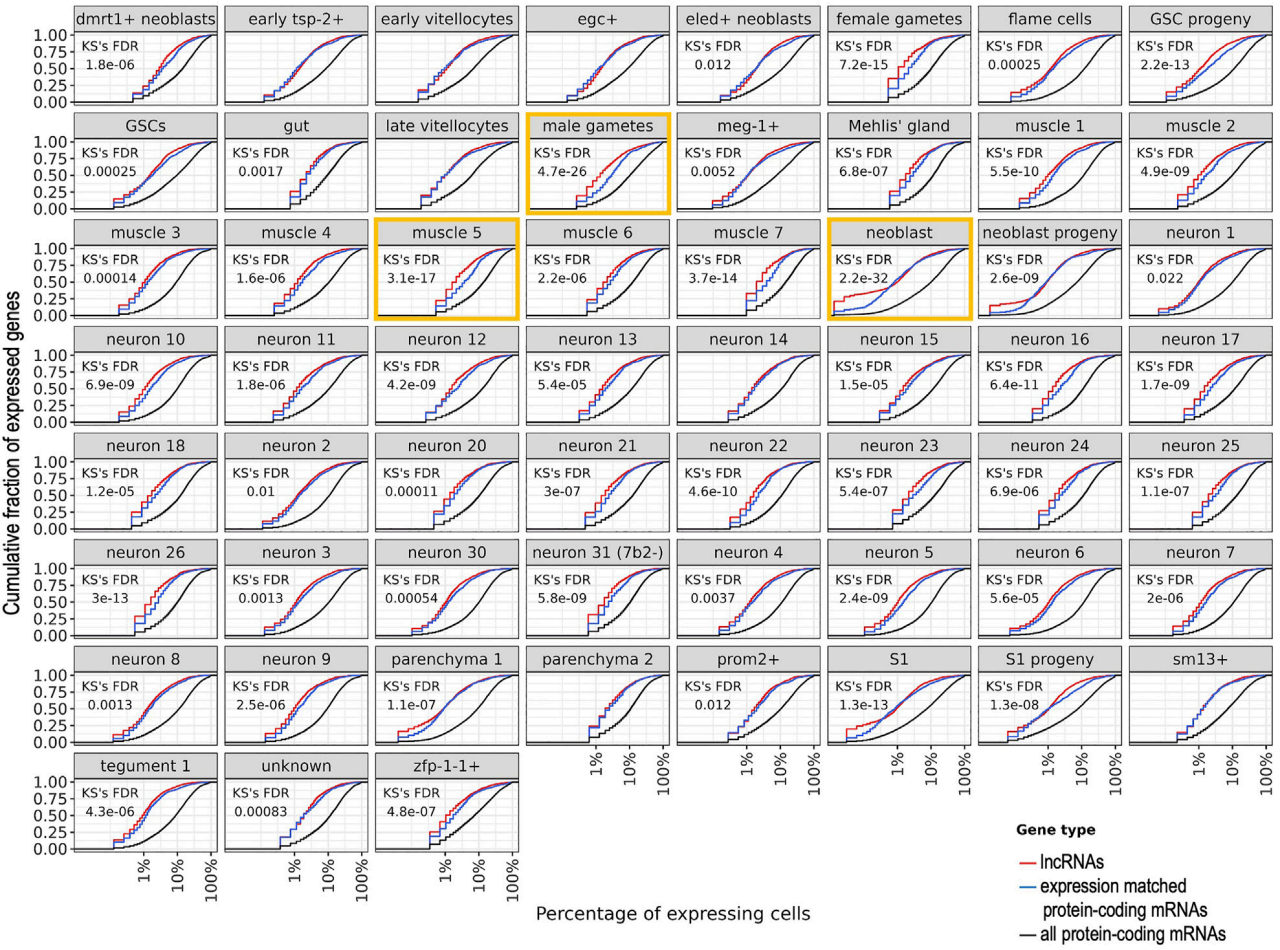
Heterogeneity of expression of all lncRNAs or mRNAs that were detected in a given cluster of cells. For each cluster named at the top of each panel, the cumulative fraction of all lncRNAs or mRNAs that were detected as expressed in the cluster (*y*-axis) is shown as a function of the percentage of cells in which the lncRNAs or the mRNAs were detected (*x*-axis). For each cluster, the red curve shows the detected lncRNAs, the blue curve shows the set of mRNAs detected with expression levels in the same range as that of the lncRNAs, and the black curve shows the complete set of mRNAs detected in the cluster. The Kolmogorov-Smirnov (KS) statistical test False Discovery Rate (FDR) is shown for the comparison between the lncRNAs and the expression-matched set of mRNAs; in the seven panels where no KS FDR is shown, no statistical difference was found (FDR >0.05). The three clusters with the most significant differences (lowest FDRs) are marked with orange rectangles. Nine clusters with less than 100 cells each were excluded from this analysis.

To evaluate the lncRNAs expression heterogeneity across cells, we then computed the cumulative fraction of all lncRNAs or mRNAs that were detected in one cluster as a function of the percentage of cells in which the lncRNAs or the mRNAs were detected (Figure 9). In all but seven clusters, a statistically significant lower percentage of cells were detected as expressing lncRNAs compared to cells expressing the set of mRNAs of similar expression levels (FDR = 0.022 to 2.2 × 10^–32^, Kolmogorov-Smirnov KS test). The top three clusters with higher heterogeneity of expression of lncRNAs compared to the set of expression-matched mRNAs were neoblasts, male gametes, and muscle 5 (Figure 9, orange rectangles). Of note, half of the lncRNAs expressed in one given cluster were detected in up to 1–3% of cells (Figure 9, red curves), whereas the mRNAs of similar expression levels were detected in a significantly higher percentage of cells (Figure 9, blue curves are significantly shifted to the right compared to the red curves), and analyzing the complete set of mRNAs expressed in one given cluster, half of the mRNAs were detected in up to 10–20% of cells (Figure 9, black curves). Because a considerable number of lncRNAs were detected in 1–3% of cells, we again searched for lncRNAs exclusively expressed in a single cluster, this time in at least 1% of cells, using a stringent requirement of exclusive expression, namely that the lncRNA was not expressed in more than 1% of cells in any other cluster (Supplementary Figure S6). Again, male gametes cluster has the highest number of exclusive lncRNAs, followed by late vitellocytes and female gametes (Supplementary Figure S6); the list of all lncRNAs and protein-coding mRNAs expressed in at least 1% of cells exclusively in one cluster is given in Supplementary Table S7. Interestingly, the exclusive protein-coding mRNAs detected in the gut cluster include Cathepsins B, L and S, Saposin B domain-containing protein, Prosaposin, Phospholipase A, and Sphingomyelin phosphodiesterase 2, whereas the exclusive protein-coding mRNAs detected in the oesophageal gland cluster include MEGs 4, 9, 11 and 32.2, Annexin, Cystatin, and Natterin-4 (Supplementary Table S7). These lncRNAs and mRNAs might play specific roles in a fraction of cells in those clusters.

Confirming that lncRNAs expression is heterogeneous across cells, we observed that only 626 different lncRNAs (8%) were non-exclusively detected in at least 10% of cells in any cluster, when compared with all 8023 different lncRNAs detected with scRNA-seq across all *S. mansoni* cells. For comparison, 7563 Smp protein-coding messages (81%) were detected in at least 10% of cells in any cluster, compared with all 9388 different Smps detected with scRNA-seq across all *S. mansoni* cells.

Supplementary Table S8 shows the number of lncRNAs and of Smp protein-coding genes not exclusively expressed in any cluster and detected as expressed in 10, 25, 50, 75 or 95% of cells in each cluster. The top 5 clusters with the highest numbers of lncRNAs (in at least 10% of the cells) were neuron 19, dmrt1^+^ neoblasts, male gametes, eled^+^ neoblasts, GSC progeny; note that the same lncRNA is counted multiple times, when it is expressed in multiple clusters.

## 4 Discussion

In this work we have re-analyzed publicly available scRNA-seq data obtained from *S. mansoni* adult male and immature and mature adult female, showing that lncRNAs are differentially expressed across different single-cell clusters. Whole-mount *in situ* hybridization and double fluorescence *in situ* hybridization confirmed the localization of most of the single-cell cluster lncRNA markers in specific adult *S. mansoni* tissues. Our re-analyses detected the expression of 8023 lncRNAs, 79.4% of all 10,110 lncRNAs known in *S. mansoni* (Maciel et al., 2019). Further scRNA-seq with deeper coverage and including other life-cycle stages may reveal a more detailed pattern of expression of lncRNAs possibly involved in parasite development and homeostasis.

Here, our approach was to use the set of protein-coding gene markers of single-cell clusters that had been extensively characterized in adult *S. mansoni* (Wendt et al., 2020) to probe lncRNAs tissue co-localization. For this, we relied on the well-documented strategy of transfer of cell clusters ID annotations from one set of scRNA-seq data used as reference, to another query set (Stuart et al., 2019). We found that in 91% of the previously annotated clusters, the great majority of cells (66–100%) were re-mapped to the same original clusters, attesting to the robustness of the transfer method (Stuart et al., 2019). In fact, only two original clusters were depleted by over 50% of their cells, and the cells were transferred to closely related clusters, namely neuron 19, where 89.4% of the cells were transferred to neuron 8 cluster, and hes2^+^, where 94.7% of the cells were transferred to the neoblast progeny cluster. Two factors may have played a role in the transfer of cells to different clusters. First, the expression level of genes may have changed because the raw scRNA-seq reads have been re-mapped to the genome and counted with the STARsolo tool, rather than with CellRanger used in the original paper (Wendt et al., 2020); STARsolo uses a different algorithm to quantify gene expression, which results in a higher number of recovered cells compared with CellRanger (Kaminow et al., 2021). We checked the similarity of the two sets of original and re-mapped expression data of protein-coding genes and found that they are highly correlated (0.972–1.0, Pearson correlation), thus ruling out a major impact of gene expression counting on the discrepant re-mapping of cells. A second factor could be the similarity between the overall expression profiles among certain clusters, which may affect the identification of proper anchor cells that are used for guiding the assignment of cells to clusters (Stuart et al., 2019). Despite these factors, cells were mostly transferred to related clusters, thus not impairing the ability to use the established protein-coding gene markers as a tool to determine co-localization of lncRNAs in the parasite tissues.

An average of 1.65 lncRNA isoforms per lncRNA gene in *S. mansoni* was identified in our previous work using RNA-seq libraries from whole worms at different stages, from isolated tissues, from cell populations, and from single-cells (Maciel et al., 2019). Among the different isoforms in a gene locus, there are alternate transcription start sites (TSSs), alternate use of exons, and exon skipping; exon splice sites identified for all these lncRNA isoforms have canonical GU/AG splicing acceptor/donor pairs (Maciel et al., 2019), thus making them bona-fide alternatively spliced messages in *S. mansoni*, in analogy to the large number of lncRNA isoforms in animals (Ulitsky, 2016); of note, no systematic analysis of the functional impact of lncRNA isoforms on *S. mansoni* biology has been documented. Here, we have selected one female gametes lncRNA marker, G16045 with 12 transcript isoforms, and we used two probes that encompass two groups of isoforms, with two different TSSs and two different last exons. We observed that one group of isoforms was expressed only in the ovary, while the other was expressed in the ovary and in the testis, giving support for a tissue-specific use of different lncRNA isoforms in *S. mansoni*. Further exploration of the wide occurrence of lncRNAs alternative splicing is warranted.

In *S. mansoni*, lncRNA knock-down with dsRNA caused important phenotypic changes such as a decreased worm viability and impaired oviposition (Silveira et al., 2022). We propose that the lncRNAs identified here as single-cell cluster markers might be good candidates to be targeted and possibly interfere with adult schistosomes homeostasis, especially those of tegument and gametes lineages. We corroborate with findings from the literature, which show that gamete lineages in animals and plants are rich in lncRNAs expression (Golicz et al., 2018; Guo et al., 2018). Four gametes marker lncRNAs were confirmed with WISH, two in male gametes (out of the three tested) and two in female gametes (Figure 7). Given their conspicuous expression in the gametes, these lncRNAs might be important for fertilized egg production and can be candidate targets to be silenced and potentially disrupt the completion of the parasite life cycle.

We found that in *S. mansoni*, the tegument progenitor lineages express a high number of lncRNAs compared with other clusters. Tegument interface protects the parasite from host (Skelly and Wilson, 2006; Wendt et al., 2018). We found a female-specific lncRNA in the tegument (G29145), and 7 lncRNA markers that are expressed in meg-1^+^ and zfp-1-1^+^ clusters, two clusters that belong to the tegument lineage. With dFISH, localization of 6 lncRNA markers in the meg-1^+^, zfp-1-1^+^ and egc^+^ clusters was confirmed. They might be good candidate targets to interfere with tegumental development, thus breaking the parasite-host barrier.

LncRNAs are known in other organisms to act in the nucleus (as enhancers, histone modification modulators, and activators/inhibitors of transcription) and in the cytoplasm (by inhibition of translation) (Statello et al., 2021). Here we observed that the marker of neuron clusters and muscle 2 cluster, lncRNA G26764 (SmLNCA149530/1) is a good example of nuclear localization, in cells where the messages of neuropeptide *7b2* gene neuron marker and of *tpm2* gene muscle marker are predominantly localized in the cytoplasm (Figures 6C,G).

Interestingly, lncRNAs expression distribution across cells in a given cluster was significantly more heterogeneous than that of protein-coding mRNAs expressed at levels similar to those of the lncRNAs, for all but seven clusters, among the 59 clusters analyzed here (Figure 9). While only 626 out of 8023 lncRNAs (8%) are expressed in at least 10% of cells from a given cluster, with a median of 126 lncRNAs per cluster (1.6%), a median of 4060 mRNAs per cluster out of 9388 protein-coding mRNAs (43%) are expressed in at least 10% of cells from a given cluster (Supplementary Table S8). LncRNAs’ cell-to-cell expression heterogeneity seems to epitomize one of the fundamental properties of the lncRNA expression patterns (Lv et al., 2016; Yunusov et al., 2016). Nevertheless, one cannot rule out the possibility that the low levels of expression of a set of lncRNAs might impose difficulties in detecting them, thus resulting into some overestimation of the extent of expression heterogeneity. The significantly higher cell-to-cell expression heterogeneity of lncRNAs compared to mRNAs might be related to the fact that while proteins are expected to play basal roles that are shared between different cells in a given tissue, lncRNAs are expressed with considerably higher tissue-specificity, developmental stage-specificity, and cell-subtype specificity (Liu et al., 2016; Yunusov et al., 2016). In fact, it has recently been shown that lncRNAs are expressed with higher cell-to-cell variability than mRNAs across a wide range of expression levels in mouse fibroblasts, in mouse embryonic stem cells and in human HEK293 cells, highlighting lncRNAs with cell state-specific functions involved in cell cycle progression and apoptosis (Johnsson et al., 2022). There are intrinsic differences in transcriptional bursting kinetics between lncRNAs and mRNAs, with lncRNAs having a fourfold lower burst frequency compared to mRNAs and only a twofold decrease in burst size (Johnsson et al., 2022). Thus, the decreased expression of lncRNAs is mainly achieved through a longer duration between transcriptional bursts of expression, which accounts for a high cell-to-cell heterogeneity of lncRNAs expression (Johnsson et al., 2022). In this regard, the half-life of a class of lncRNAs has been shown in humans to be shorter than that of mRNAs (Ayupe et al., 2015). Altogether, our data is compatible with a transient, desynchronized expression of lncRNAs in a diverse population of cells from the same tissue, which calls the attention to the fact that lncRNAs with low population-level abundance might instead be expressed at high levels in a subset of individual cells within that population, where they may have important functions.

LncRNAs may act *in cis*, regulating the neighbor protein-coding genes (Rinn and Chang, 2020). Localization of a lncRNA in the genome, and identification of protein-coding gene neighbors can give clues to possible mechanisms of action. Curiously, lncRNA G39666 (SmLINC173882) neuron marker is located in an intergenic region (http://genome.verjolab.usp.br/cgi-bin/hgTracks?hgS_doLoadUrl=submit&hgS_loadUrlName=http://genome.verjolab.usp.br/folders/geneNetwork/schMan3/tracks/genes/lincRNAs/htmlPage/Morales-VicenteG39666publicLocus.txt), between neural-cadherin (Smp_306,450.1) and an uncharacterized protein (Smp_084010.1) conserved in helminths. Because expression of the latter was strongly detected in almost all neuron clusters (http://verjolab.usp.br:8081/cluster_view/Smp-084010), further studies could elucidate a possible regulatory function of the lncRNA on the expression of this protein coding gene.

In conclusion, in this study we provide a comprehensive view of the expression of lncRNAs in the different cell types of adult *S. mansoni*, paving the way for functional studies of lncRNAs as potential regulators of the parasite homeostasis.

## Data Availability

The datasets presented in this study can be found in online repositories. The names of the repository/repositories and accession number(s) can be found in the article/Supplementary Material.
