# Supplementary material for: Single-cell RNA-seq analyses show that long non-coding RNAs are conspicuously expressed in Schistosoma mansoni gamete and tegument progenitor cell populations
Source: Front Genet. 2022 Sep 20;13:924877. doi: 10.3389/fgene.2022.924877 (PMC9531161; doi:10.3389/fgene.2022.924877)
Supplement: Supplementary file 1 [file Image5.pdf]

Figure S5

A

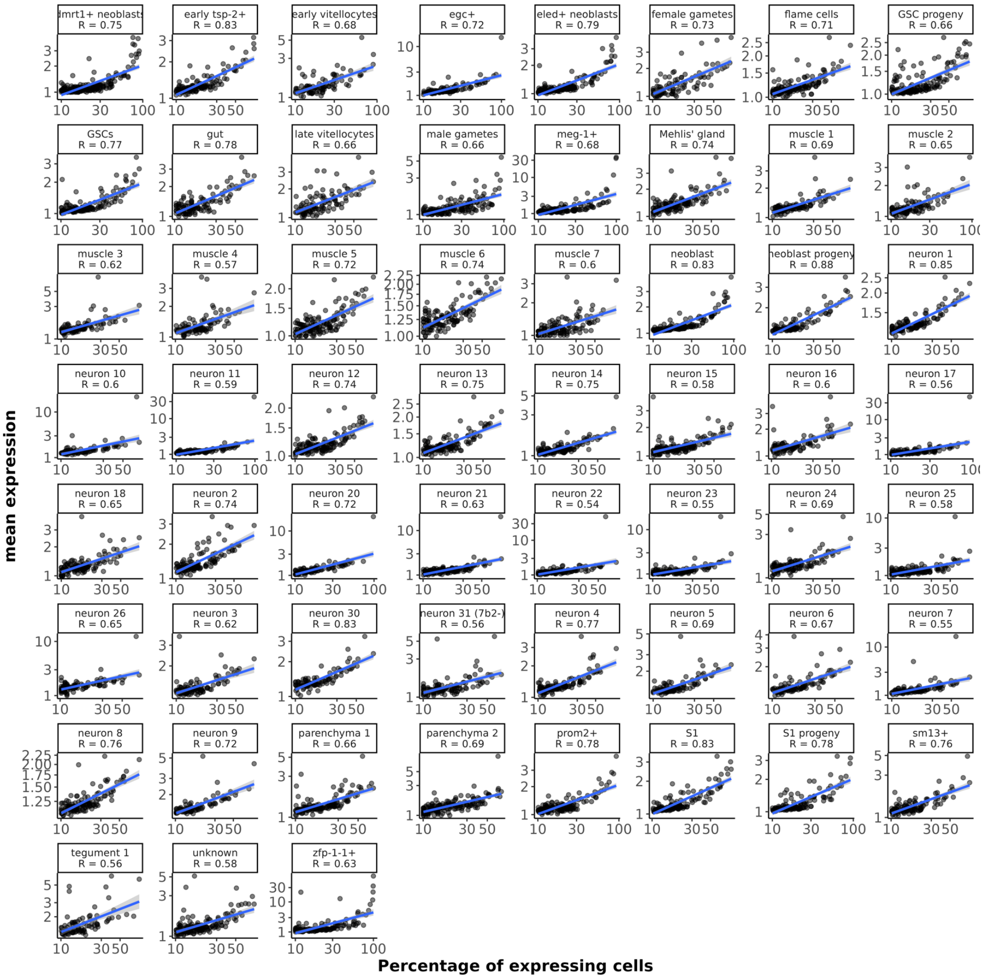
dmrt1+ neoblasts R = 0.75
early tsp-2+ R = 0.83
early vitellocytes R = 0.68
egc+ R = 0.72
eled+ neoblasts R = 0.79
female gametes R = 0.73
flame cells R = 0.71
GSC progeny R = 0.66
GSCs R = 0.77
gut R = 0.78
late vitellocytes R = 0.66
male gametes R = 0.66
meg-1+ R = 0.68
Mehlis' gland R = 0.74
muscle 1 R = 0.69
muscle 2 R = 0.65
muscle 3 R = 0.62
muscle 4 R = 0.57
muscle 5 R = 0.72
muscle 6 R = 0.74
muscle 7 R = 0.6
neoblast R = 0.83
neoblast progeny R = 0.88
neuron 1 R = 0.85
neuron 10 R = 0.6
neuron 11 R = 0.59
neuron 12 R = 0.74
neuron 13 R = 0.75
neuron 14 R = 0.75
neuron 15 R = 0.58
neuron 16 R = 0.6
neuron 17 R = 0.56
neuron 18 R = 0.65
neuron 2 R = 0.74
neuron 20 R = 0.72
neuron 21 R = 0.63
neuron 22 R = 0.54
neuron 23 R = 0.55
neuron 24 R = 0.69
neuron 25 R = 0.58
neuron 26 R = 0.65
neuron 3 R = 0.62
neuron 30 R = 0.83
neuron 31 (7b2-) R = 0.56
neuron 4 R = 0.77
neuron 5 R = 0.69
neuron 6 R = 0.67
neuron 7 R = 0.55
neuron 8 R = 0.76
neuron 9 R = 0.72
parenchyma 1 R = 0.66
parenchyma 2 R = 0.69
prom2+ R = 0.78
S1 R = 0.83
S1 progeny R = 0.78
sm13+ R = 0.76
tegument 1 R = 0.56
unknown R = 0.58
zfp-1-1+ R = 0.63
mean expression
Percentage of expressing cells

B

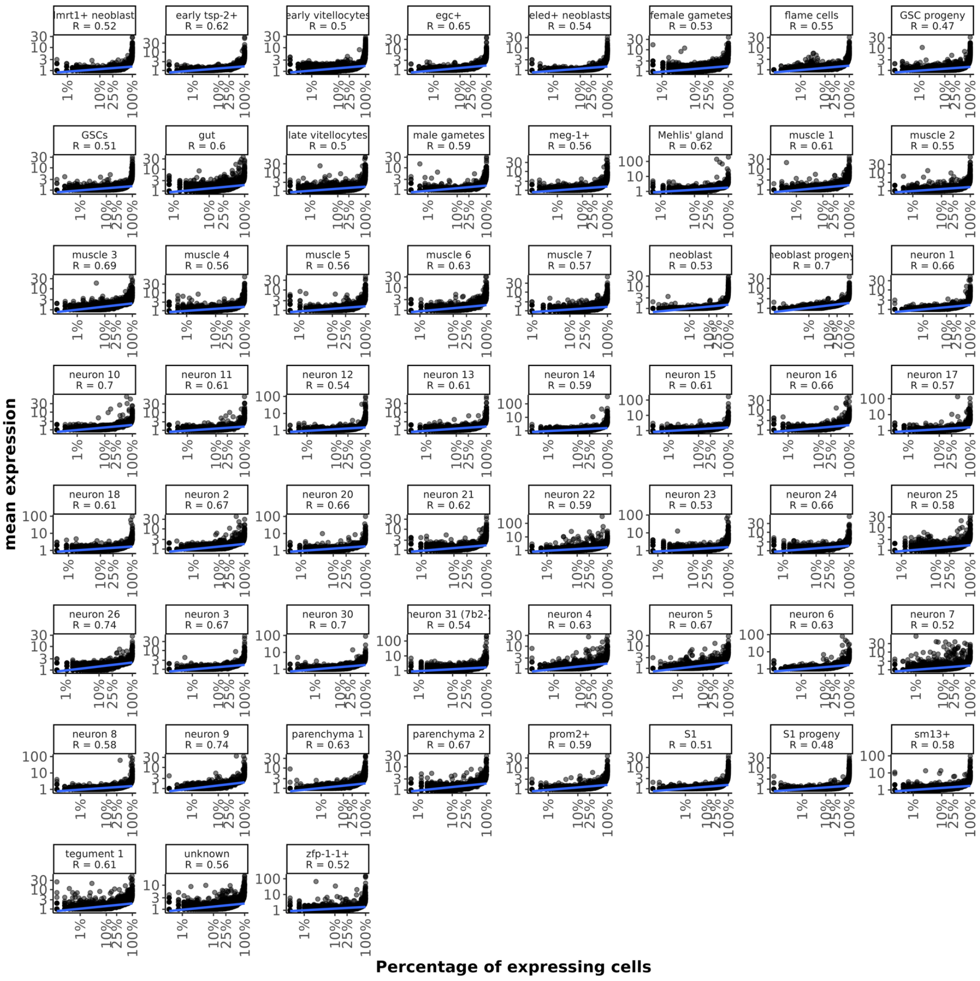
dmrt1+ neoblast R = 0.52
early tsp-2+ R = 0.62
early vitellocytes R = 0.5
egc+ R = 0.65
eled+ neoblasts R = 0.54
female gametes R = 0.53
flame cells R = 0.55
GSC progeny R = 0.47
GSCs R = 0.51
gut R = 0.6
late vitellocytes R = 0.5
male gametes R = 0.59
meg-1+ R = 0.56
Mehlis' gland R = 0.62
muscle 1 R = 0.61
muscle 2 R = 0.55
muscle 3 R = 0.69
muscle 4 R = 0.56
muscle 5 R = 0.56
muscle 6 R = 0.63
muscle 7 R = 0.57
neoblast R = 0.53
neoblast progeny R = 0.7
neuron 1 R = 0.66
neuron 10 R = 0.7
neuron 11 R = 0.61
neuron 12 R = 0.54
neuron 13 R = 0.61
neuron 14 R = 0.59
neuron 15 R = 0.61
neuron 16 R = 0.66
neuron 17 R = 0.57
neuron 18 R = 0.61
neuron 2 R = 0.67
neuron 20 R = 0.66
neuron 21 R = 0.62
neuron 22 R = 0.59
neuron 23 R = 0.53
neuron 24 R = 0.66
neuron 25 R = 0.58
neuron 26 R = 0.74
neuron 3 R = 0.67
neuron 30 R = 0.7
neuron 31 (7b2- R = 0.54
neuron 4 R = 0.63
neuron 5 R = 0.67
neuron 6 R = 0.63
neuron 7 R = 0.52
neuron 8 R = 0.58
neuron 9 R = 0.74
parenchyma 1 R = 0.63
parenchyma 2 R = 0.67
prom2+ R = 0.59
S1 R = 0.51
S1 progeny R = 0.48
sm13+ R = 0.58
tegument 1 R = 0.61
unknown R = 0.56
zfp-1-1+ R = 0.52
mean expression
Percentage of expressing cells

Supplementary Figure S5 – Correlation between the expression level of genes and the percentage of cells of a given cluster in which those genes were detected. In (**A**) the expressed lncRNAs are shown, and in (**B**) the protein-coding mRNAs. For each cluster, named at the top of each panel, the mean expression of each gene (y-axis) is plotted as a function of the percentage of cells in the cluster that are detected as expressing that gene (x-axis). The correlation coefficient R is shown below the name of the cluster. Nine clusters with less than 100 cells each were excluded from this analysis.
