# Supplementary material for: Single-cell RNA-seq analyses show that long non-coding RNAs are conspicuously expressed in Schistosoma mansoni gamete and tegument progenitor cell populations
Source: Front Genet. 2022 Sep 20;13:924877. doi: 10.3389/fgene.2022.924877 (PMC9531161; doi:10.3389/fgene.2022.924877)
Supplement: Supplementary file 2 [file Image6.pdf]

Figure S6

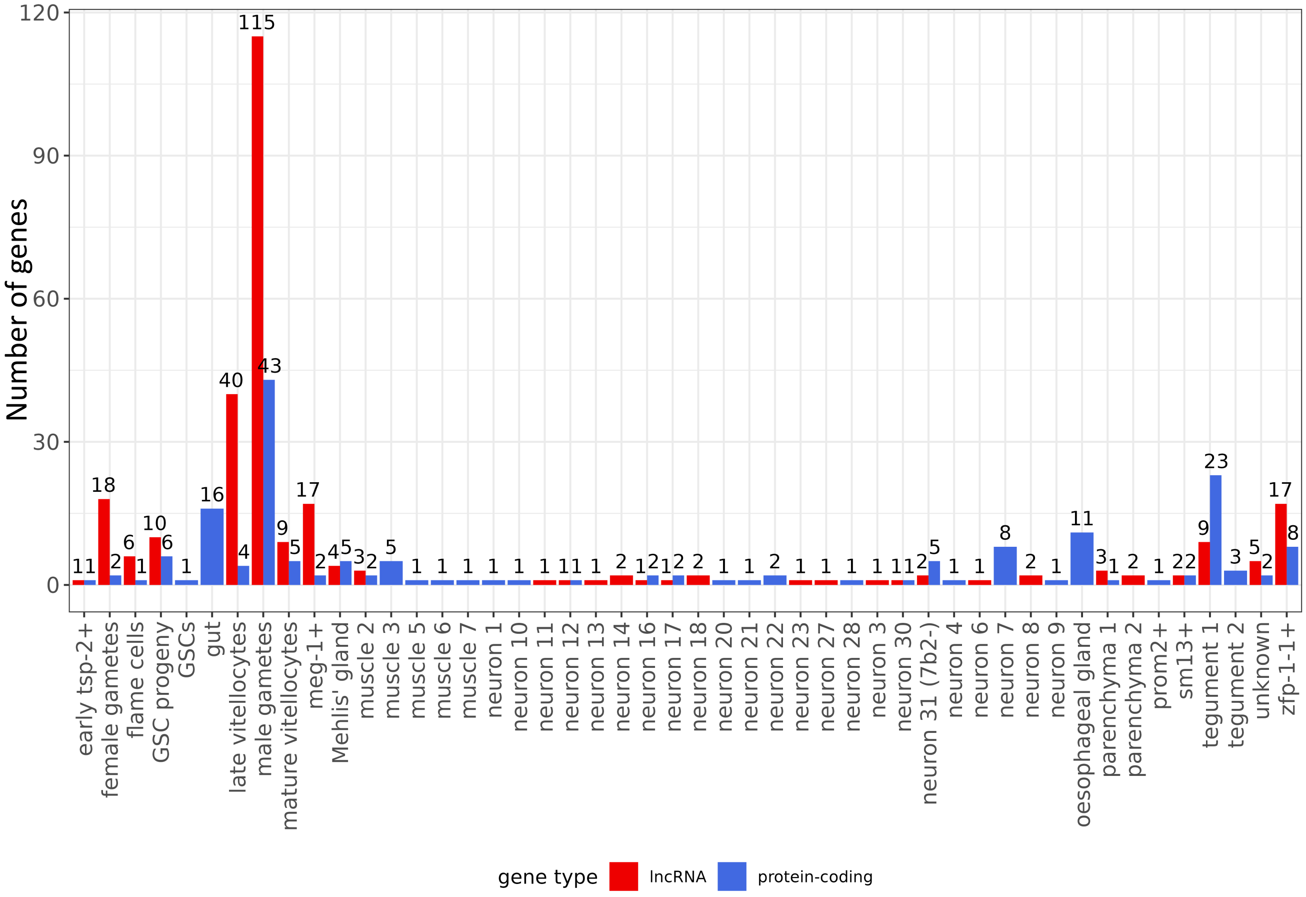

Supplementary Figure S6 – Number of lncRNAs and protein-coding mRNAs exclusively expressed in at least 1% of the cells in only one cluster. The red bars show the number of lncRNAs exclusively detected as expressed in at least 1% of the cells in the single cluster indicated in the x-axis, and not detected in more than 1% of the cells in any other cluster. For comparison, the blue bars show the number of protein-coding mRNAs with the same cluster expression patterns. Genes expressed in less than 5 cells in a cluster were excluded from this analysis.
