# Supplementary material for: Single-cell RNA-seq analyses show that long non-coding RNAs are conspicuously expressed in Schistosoma mansoni gamete and tegument progenitor cell populations
Source: Front Genet. 2022 Sep 20;13:924877. doi: 10.3389/fgene.2022.924877 (PMC9531161; doi:10.3389/fgene.2022.924877)
Supplement: Supplementary file 3 [file Image4.pdf]

Figure S4

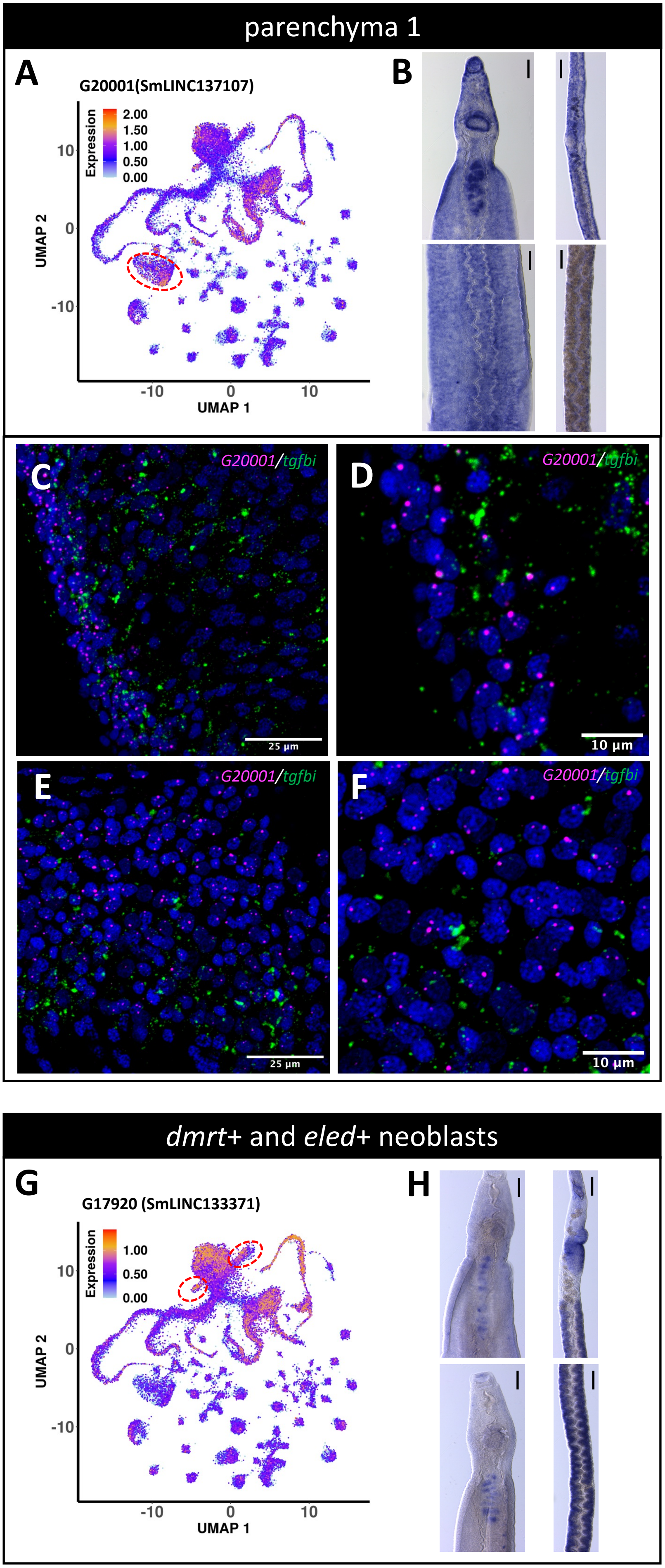
parenchyma 1
A
G20001(SmLINC137107)
Expression
2.00
1.50
1.00
0.50
0.00
UMAP 2
UMAP 1
B
C
G20001/tgfbi
25 µm
D
G20001/tgfbi
10 µm
E
G20001/tgfbi
25 µm
F
G20001/tgfbi
10 µm
dmrt+ and eled+ neoblasts
G
G17920 (SmLINC133371)
Expression
1.00
0.50
0.00
UMAP 2
UMAP 1
H

Supplementary Figure S4 – Two lncRNA candidate markers were not validated in the corresponding clusters. (**A**) UMAP plot of lncRNA G20001 marker of parenchyma 1 cluster. (**B**) WISH with lncRNA G20001 in the head [left, top] and trunk [left, bottom] of a male, and in the ovary region [right, top] and vitellaria [right, bottom] of a female. (**C to F**) Double FISH in male trunk with lncRNA G20001 and the general parenchyma 1 marker gene *tgfbi*. (**G**) UMAP plot of lncRNA G17920 marker of *dmrt*+ and *eled*+ neoblast clusters. (**H**) WISH with lncRNA G17920 in the head [left, top and bottom] of two males, and in the ovary region [right, top] and vitellaria [right, bottom] of a female. UMAP plots are colored by gene expression (blue = low, red = high) and the scale represents log10(UMIs+1). The regions enclosed by the red dashed lines indicate the location of the relevant parenchyma 1 [A] and *dmrt*+ and *eled*+ neoblast [G] clusters on the UMAP plots. WISH scale bars are 100 μm.
