# Supplementary material for: Single-cell RNA-seq analyses show that long non-coding RNAs are conspicuously expressed in Schistosoma mansoni gamete and tegument progenitor cell populations
Source: Front Genet. 2022 Sep 20;13:924877. doi: 10.3389/fgene.2022.924877 (PMC9531161; doi:10.3389/fgene.2022.924877)
Supplement: Supplementary file 4 [file Image2.pdf]

Figure S2

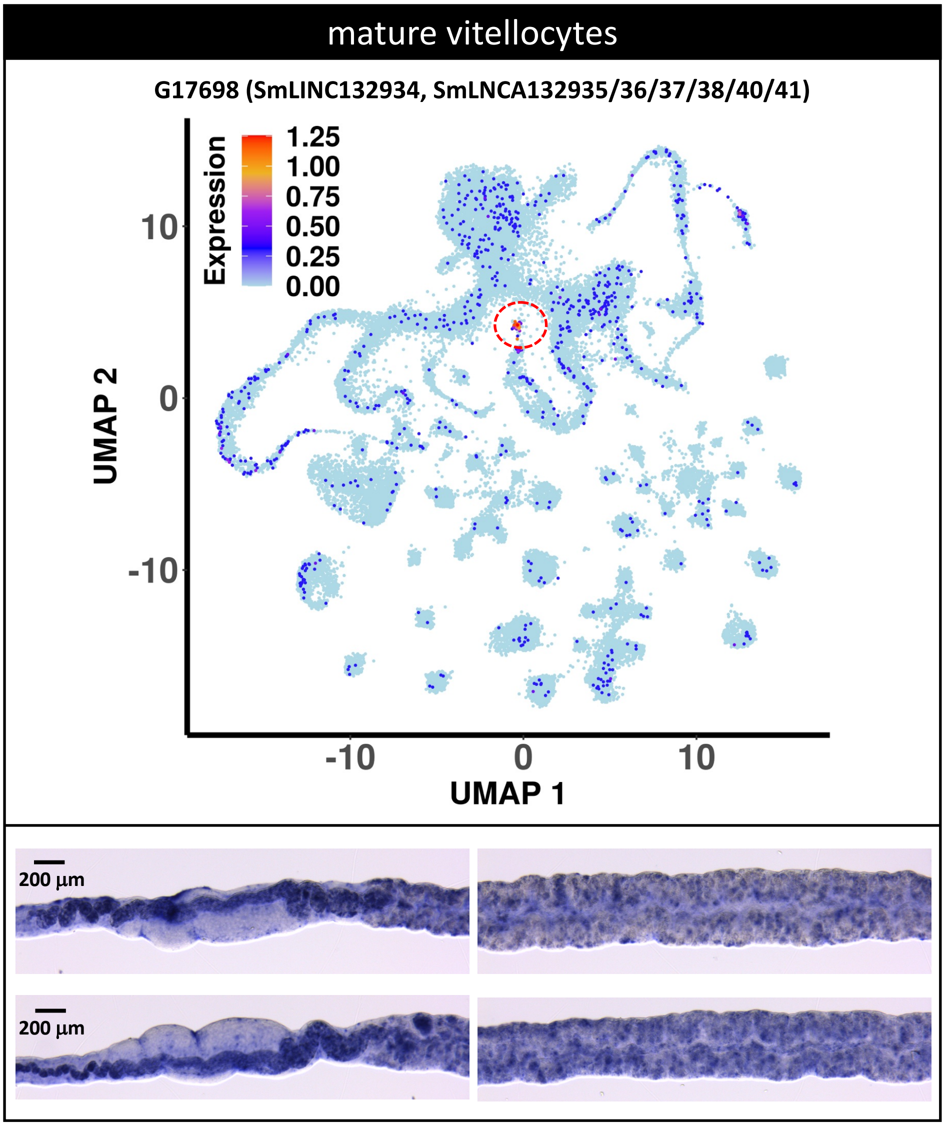

Supplementary Figure S2 – lncRNA marker of mature vitellocytes cluster. UMAP plot (top) of lncRNA G17698 marker of mature vitellocytes cluster. WISH (bottom) with lncRNA G17698 in the ovary region of two females [left, top and bottom] and in the vitellaria [right, top and bottom]. UMAP plots are colored by gene expression (blue = low, red = high) and the scale represents log10(UMIs+1). The region enclosed by the red dashed line indicates the location of the relevant mature vitellocytes cluster on the UMAP plot.
