# Supplementary material for: Single-cell RNA-seq analyses show that long non-coding RNAs are conspicuously expressed in Schistosoma mansoni gamete and tegument progenitor cell populations
Source: Front Genet. 2022 Sep 20;13:924877. doi: 10.3389/fgene.2022.924877 (PMC9531161; doi:10.3389/fgene.2022.924877)
Supplement: Supplementary file 5 [file Image3.pdf]

Figure S3

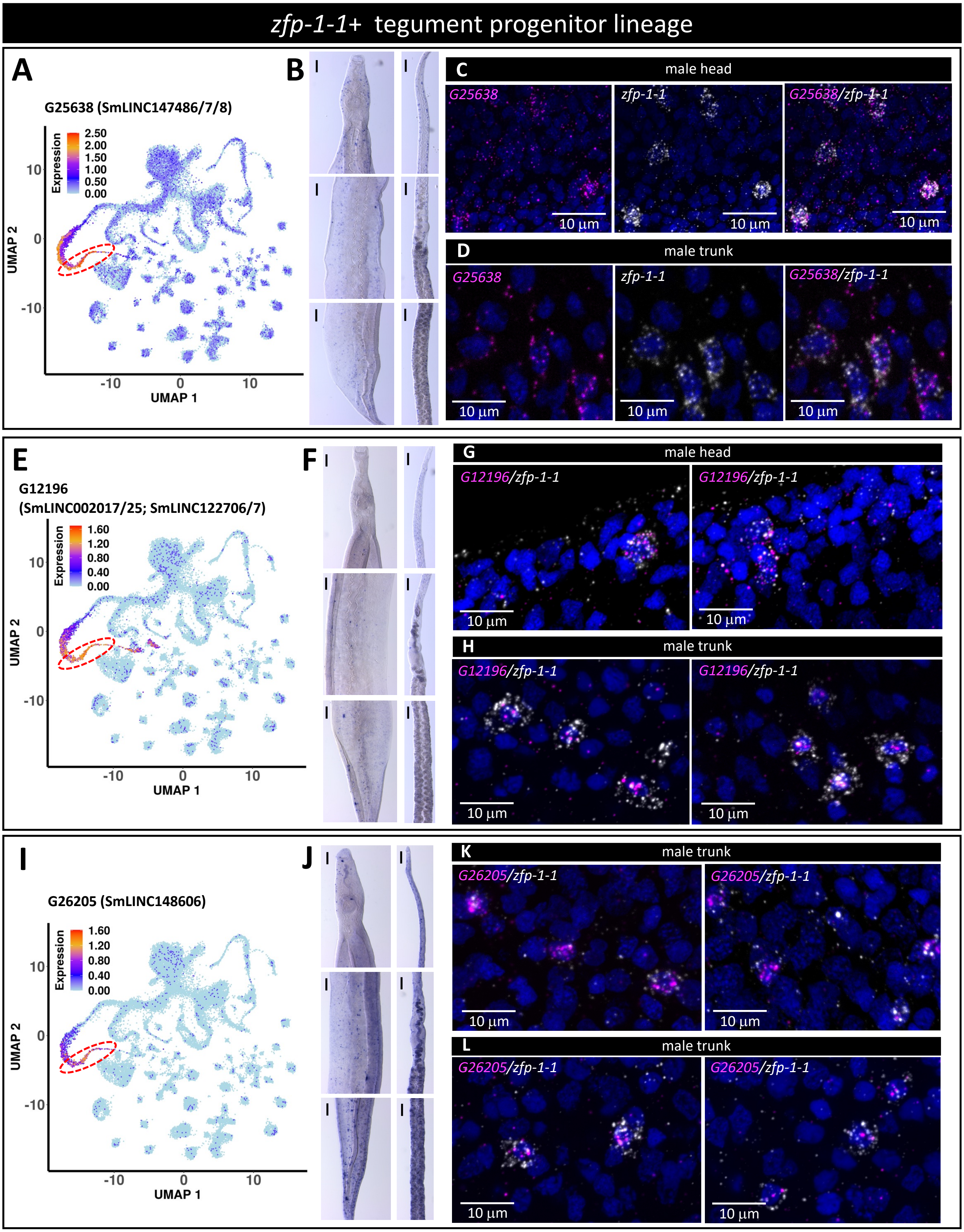

zfp-1-1+ tegument progenitor lineage
A
G25638 (SmLINC147486/7/8)
Expression
2.50
2.00
1.50
1.00
0.50
0.00
UMAP 2
UMAP 1
B
C
male head
G25638
zfp-1-1
G25638/zfp-1-1
10 µm
D
male trunk
E
G12196
(SmLINC002017/25; SmLINC122706/7)
1.60
1.20
0.80
0.40
0.00
F
G
G12196/zfp-1-1
H
I
G26205 (SmLINC148606)
J
K
G26205/zfp-1-1
L

Supplementary Figure S3 – Additional lncRNA markers of *zfp-1-1*+ tegument progenitor cluster. (**A, E, I**) UMAP plot of the indicated lncRNA marker of tegument progenitor lineages. (**B, F, J**) WISH with the indicated lncRNA in a male [left] and a female [right] head [top], trunk [middle] and tail [bottom]. (**C, G**) Double FISH in male head with the indicated lncRNA and the general tegument progenitor marker gene *zfp-1-1*. (**D, H, K, L**) Double FISH in male trunk with the indicated lncRNA and the general tegument progenitor marker gene *zfp-1-1*. UMAP plots are colored by gene expression (blue = low, red = high) and the scale represents log10(UMIs+1). The regions enclosed by the red dashed lines indicate the location of the relevant *zfp-1-1*+ cluster on the UMAP plots. WISH scale bars are 100 μm.
