# Supplementary material for: Single-cell RNA-seq analyses show that long non-coding RNAs are conspicuously expressed in Schistosoma mansoni gamete and tegument progenitor cell populations
Source: Front Genet. 2022 Sep 20;13:924877. doi: 10.3389/fgene.2022.924877 (PMC9531161; doi:10.3389/fgene.2022.924877)
Supplement: Supplementary file 6 [file Image1.pdf]

Figure S1

A

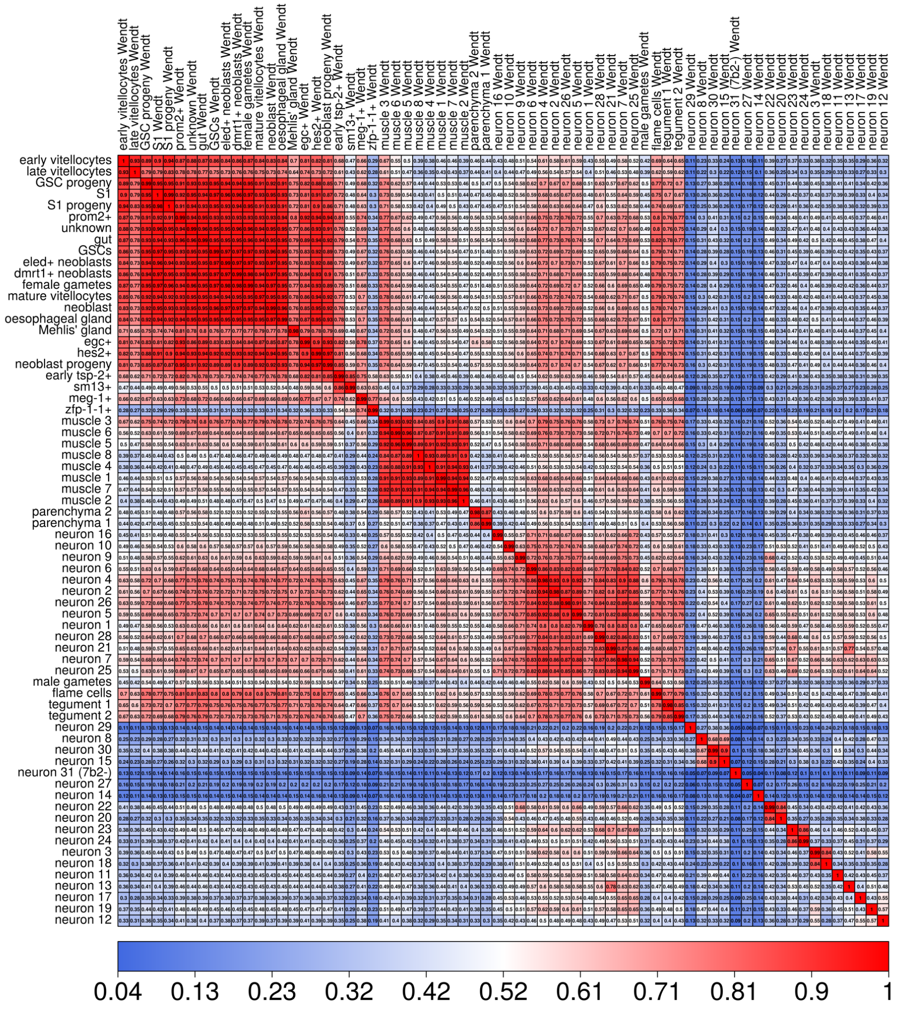
early vitellocytes
late vitellocytes
GSC progeny
S1
S1 progeny
prom2+
unknown
gut
GSCs
eled+ neoblasts
dmrt1+ neoblasts
female gametes
mature vitellocytes
neoblast
oesophageal gland
Mehlis' gland
egc+
hes2+
neoblast progeny
early tsp-2+
sm13+
meg-1+
zfp-1-1+
muscle 3
muscle 6
muscle 5
muscle 8
muscle 4
muscle 1
muscle 7
muscle 2
parenchyma 2
parenchyma 1
neuron 16
neuron 10
neuron 9
neuron 6
neuron 4
neuron 2
neuron 26
neuron 5
neuron 1
neuron 28
neuron 21
neuron 7
neuron 25
male gametes
flame cells
tegument 1
tegument 2
neuron 29
neuron 8
neuron 30
neuron 15
neuron 31 (7b2-)
neuron 27
neuron 14
neuron 22
neuron 20
neuron 23
neuron 24
neuron 3
neuron 18
neuron 11
neuron 13
neuron 17
neuron 19
neuron 12
0.04
0.13
0.23
0.32
0.42
0.52
0.61
0.71
0.81
0.9
1

B

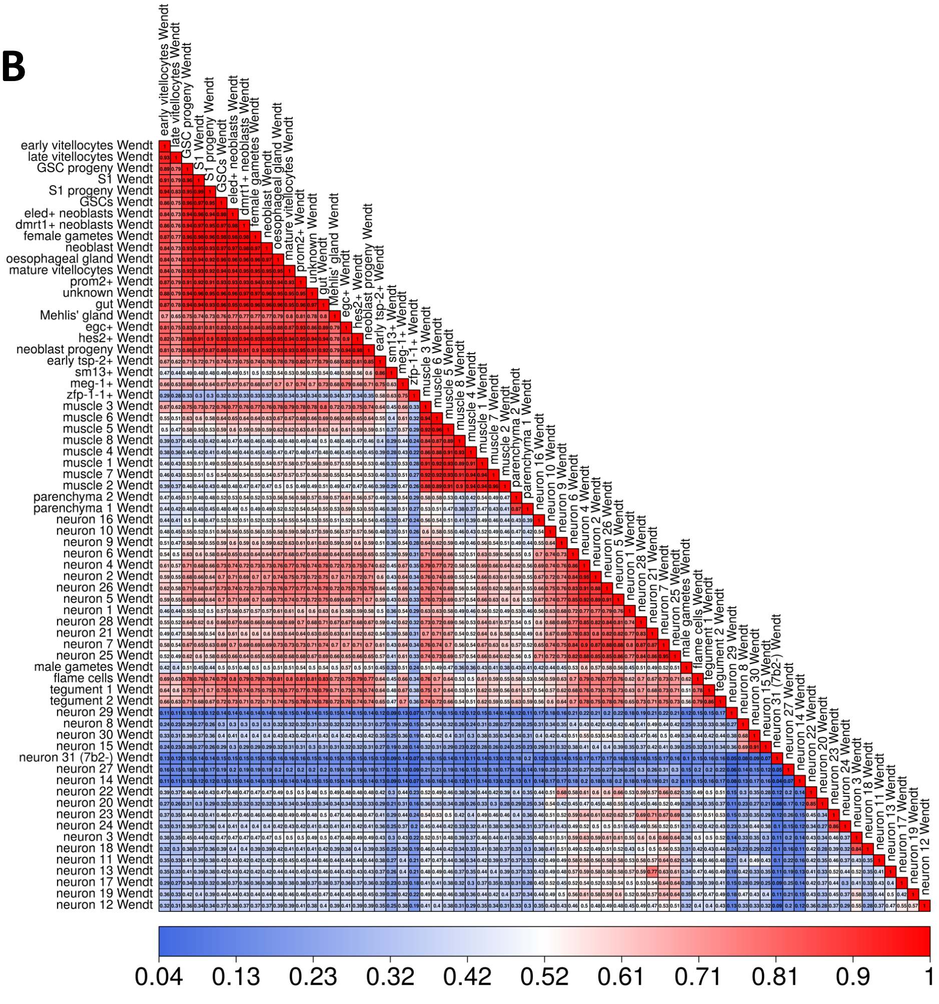
early vitellocytes Wendt
late vitellocytes Wendt
GSC progeny Wendt
S1 Wendt
S1 progeny Wendt
GSCs Wendt
eled+ neoblasts Wendt
dmrt1+ neoblasts Wendt
female gametes Wendt
neoblast Wendt
oesophageal gland Wendt
mature vitellocytes Wendt
prom2+ Wendt
unknown Wendt
gut Wendt
Mehlis' gland Wendt
egc+ Wendt
hes2+ Wendt
neoblast progeny Wendt
early tsp-2+ Wendt
sm13+ Wendt
meg-1+ Wendt
zfp-1-1+ Wendt
muscle 3 Wendt
muscle 6 Wendt
muscle 5 Wendt
muscle 8 Wendt
muscle 4 Wendt
muscle 1 Wendt
muscle 7 Wendt
muscle 2 Wendt
parenchyma 2 Wendt
parenchyma 1 Wendt
neuron 16 Wendt
neuron 10 Wendt
neuron 9 Wendt
neuron 6 Wendt
neuron 4 Wendt
neuron 2 Wendt
neuron 26 Wendt
neuron 5 Wendt
neuron 1 Wendt
neuron 28 Wendt
neuron 21 Wendt
neuron 7 Wendt
neuron 25 Wendt
male gametes Wendt
flame cells Wendt
tegument 1 Wendt
tegument 2 Wendt
neuron 29 Wendt
neuron 8 Wendt
neuron 30 Wendt
neuron 15 Wendt
neuron 31 (7b2-) Wendt
neuron 27 Wendt
neuron 14 Wendt
neuron 22 Wendt
neuron 20 Wendt
neuron 23 Wendt
neuron 24 Wendt
neuron 3 Wendt
neuron 18 Wendt
neuron 11 Wendt
neuron 13 Wendt
neuron 17 Wendt
neuron 19 Wendt
neuron 12 Wendt
0.04
0.13
0.23
0.32
0.42
0.52
0.61
0.71
0.81
0.9
1

Supplementary Figure S1 – Correlation coefficient between the median expression of clusters in the reference and query matrices. Pearson correlation between the median expression of genes in the cells from the indicated clusters is shown in (**A**) for the comparison between the query matrix generated in the present work that includes lncRNAs and protein-coding genes and the Wendt et al. (2020) reference matrix with protein-coding genes only; and in (**B**) for the comparison between clusters of the Wendt et al. (2020) reference matrix.
